# Visual hermeneutics as a tool to introduce empathy and core physician attributes in doctor-patient relationship for first-year medical undergraduate students

**DOI:** 10.1186/s12909-025-06742-6

**Published:** 2025-01-29

**Authors:** Sushma Prabhath, Uma Kulkarni, Vani Lakshmi R., Bikash Patra, Eshwari K., Divya Arvind Prabhu, Kirtana R. Nayak

**Affiliations:** 1 Department of Anatomy, Kasturba Medical College, Manipal, Manipal Academy of Higher Education, Manipal, Karnataka 576104 India; 2 Department of Ophthalmology, Yenepoya Medical College, Faculty, Centre for Ethics, Yenepoya University, Mangalore, India; 3 Department of Data Science, Prasanna School of Public Health, Manipal Academy of Higher Education, Manipal, Karnataka 576104 India; 4 Second Year Undergraduate Medical Student, Kasturba Medical College, Manipal, Manipal Academy of Higher Education, Manipal, Karnataka 576104 India; 5 Department of Community Medicine, Kasturba Medical College, Manipal Academy of Higher Education, Manipal, Karnataka 576104 India; 6 Department of Physiology, Kasturba Medical College, Manipal, Manipal Academy of Higher Education, Manipal, Karnataka 576104 India; 7 Department of Medical Education, Kasturba Medical College, Manipal, Manipal Academy of Higher Education, Manipal, Karnataka 576104 India

**Keywords:** Doctor-patient relationship, First-year medical undergraduates, AETCOM, Paintings, Visual hermeneutics

## Abstract

**Background:**

The doctor-patient relationship is essential for effective patient care, yet medical education often neglects to nurture the quality such as empathy during the initial years of training. Doctor-patient relationship is one of the modules taught in first year as part of mandatory AETCOM (Attitude, Ethics, and Communication) course in the undergraduate Indian medical curriculum. Hermeneutics, a method of interpretation, can play a vital role in introducing observational and reflective thinking skills. This study aimed to introduce empathy concepts and attributes of a good physician to first-year medical students through the use of paintings and visual hermeneutics to enhance their understanding of the doctor-patient relationship.

**Methods:**

A newly developed and validated educational module on the “Doctor-patient relationship” was administered to 250 first-year undergraduate medical students at Kasturba Medical College Manipal. The session was structured into three key steps:

**Introduction:**

Presenting Sir Luke Fildes’ famous painting, *The Doctor*.

**Self-understanding and interpretation:**

Encouraging students to analyse and interpret the painting.

**Debriefing:**

Facilitating discussions to consolidate learning. The session’s effectiveness was evaluated using the initial two levels of the Kirkpatrick evaluation model. The learners feedback regarding the usefulness of the session was captured using a validated questionnaire, while reflective writing assignments were used to explore students’ learnings from the intervention.

**Results:**

Student feedback was overwhelmingly positive, with participants expressing that the session significantly enhanced their understanding of professional qualities and empathy in the doctor-patient relationship, rating their overall learning experience at 8.63 out of 10. Students expressed that the use of painting and interactive discussions greatly enriched their capacity to connect with emotional and ethical dimensions of medical practice. Reflective writing revealed the importance of building trust, demonstrating professional conduct, and maintaining empathy and compassion in clinical interactions.

**Conclusion:**

Introducing a visual hermeneutics approach has the potential to cultivate empathy and foster a deeper understanding of the doctor-patient relationship. Introducing this approach during the formative years of medical training can nurture compassionate and virtuous conduct, reinforcing students’ commitment to delivering empathetic and competent care throughout their medical careers.

**Trial registration:**

The institutional ethics committee approved the conduct of the study [IEC180/2023].

**Supplementary Information:**

The online version contains supplementary material available at 10.1186/s12909-025-06742-6.

## Introduction

The doctor-patient relationship is the foundation of effective patient care. A good doctor-patient relationship is fundamental in providing high-quality care to patients. Both patients and doctors bring their personal beliefs, fears, and attitudes into a medical encounter, and these factors can significantly influence the dynamics and outcomes of the interaction [1]. Achieving a well-rounded approach to the patient care involves understanding patients, their illnesses, their perceptions of suffering, how they cope with illness. Teaching budding physicians, the distinction between healing and curing, along with the interaction between empathy and equanimity, is essential for building patient trust and enhancing patient satisfaction [2].

The undergraduate medical curriculum often allocates majority of its hours on teaching diseases, patient symptoms, and their management, which tends to promote a disease-centred approach rather than a patient-centred approach [3]. This focus can lead to a gap in developing skills related to understanding the patient’s experience, including their emotions, values, and context of their illness. Hermeneutics, grounded in post phenomenological philosophy, offers a framework for analysing the phenomenon of understanding human experiences through close observation and interpretation [4]. As an art and science of interpretation, hermeneutics is essential in medical practice as it involves understanding the patient’s narrative beyond the surface of clinical symptoms [5]. Applying hermeneutics means that a physician interprets not just the physical condition but also the subjective experiences, beliefs, and emotions that a patient and family communicate [6]. By adopting hermeneutic lens, doctors can see patients as whole persons and appreciate the context of their suffering which would allow them to engage with patients holistically.

While empathy allows the physicians to connect with patients at a deeper emotional level, visual hermeneutics [7, 8], the interpretation of visual art such as fine arts, can serve as a powerful tool in cultivating empathy [9]. Paintings and other forms of visual art often convey deep emotional narratives and human experiences. By engaging with these artworks, health professionals can enhance their interpretative skills, learning to discern emotions and, contexts paving way for profound understanding of human suffering. While reflecting on the themes and emotions portrayed in the visual art, learners can be trained to become more attuned to the feelings and experiences of their patients. This heightened awareness can lead to improved communication, better rapport, and a more compassionate approach to patient care [10]. Sir Luke Henry Fildes’ famous painting, *“The Doctor”* [Painting: Sir Henry Fildes, The Doctor, 1891, Source: Tate (N01522), Britain, London, Digital image ©Tate, Released under Creative Commons CC-BY-NC-ND, (3.0 Unported)], is one such visual art form in the form of painting that can be used to foray into the “Doctor-patient relationship” through visual hermeneutics [11, 12]. This painting continues to stand as a lasting representation of a Victorian general practitioner, often utilized to exemplify the attributes of an excellent physician in the present day. It embodies the image of an exemplary and committed doctor featured in various contexts. In today’s contemporary culture, it is nearly ubiquitous when assessing the merits and deficiencies of the medical profession.

The National Medical Commission (NMC) erstwhile Medical Council of India (MCI) has mandated the introduction of the ‘Attitude, Ethics and Communication’ (AETCOM) course in the UG medical curriculum of Indian schools [13]. It is based on the Graduate Medical Education Regulations (GMER)-2019 [14]. The 27 modules in AETCOM course taught from first to final professional years include various learning elements related to attitude, ethics and communication skills. The “Doctor-Patient Relationship” is one among the modules in the first professional year. The competencies addressed in this module are describing a physician’s professional qualities and demonstrating empathy in patient encounters.

We employed visual hermeneutics, using paintings, to teach competencies in the module on the “Doctor-patient relationship " for first-year medical students. The present study, therefore, aimed to develop and internally validate a teaching module on the doctor-patient relationship, focusing on the competencies of empathy and attributes of a good physician, using visual hermeneutics with paintings. We also analysed the learners’ perceptions and feedback regarding the effectiveness of this module.

## Methods

### Study design, setting, & participants

#### Study design

Interventional study-educational.

#### Study setting

Small group teaching session for first-year undergraduate medical students (UGs) at Kasturba Medical College Manipal.

#### Study participants and sample size

250 first-year medical students of the batch 2022(based on complete enumeration data).

### Inclusion & exclusion criteria

#### Inclusion criteria

All 250 first-year medical students from the 2022 batch of Kasturba Medical College, Manipal, who attended the small-group teaching session.

#### Exclusion criteria

Students who could not attend the session were excluded.

Duration of the study: One year (including data collection and analysis).

## Construction of the educational module

### Selection of the painting

**Fig. 1:**
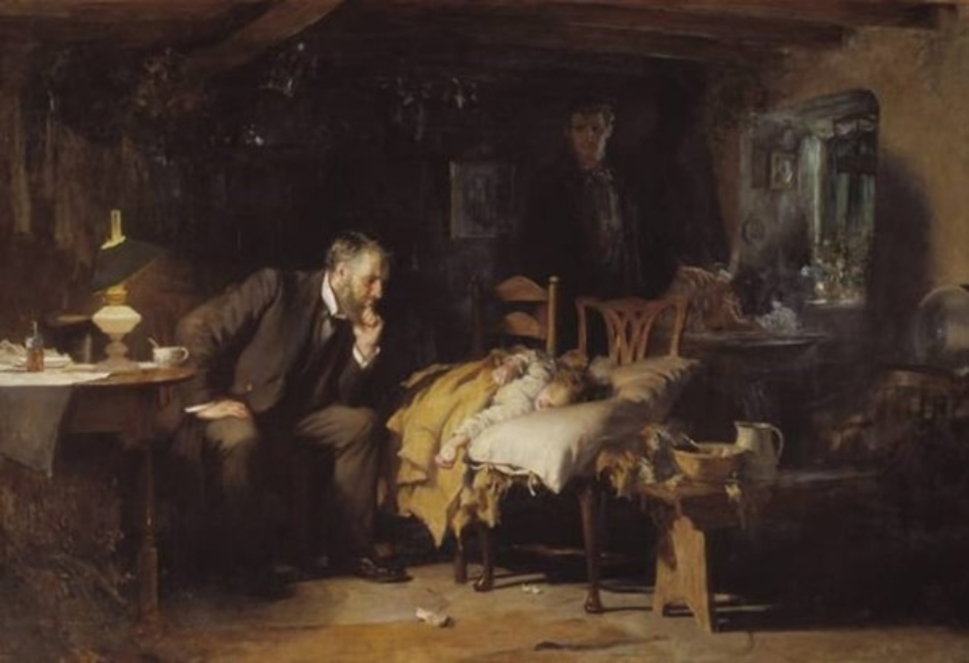
The Painting. “The Doctor” [Painting: Sir Henry Fildes, The Doctor, 1891, Source: Tate (N01522), Britain, London, Digital image ©Tate, Released under Creative Commons CC-BY-NC-ND, (3.0 Unported)]

Sir Luke Henry Fildes’ famous painting, *“The Doctor”* [Painting: Sir Henry Fildes, The Doctor, 1891, Source: Tate (N01522), Britain, London, Digital image ©Tate, released under Creative Commons CC-BY-NC-ND, (3.0 Unported)] was chosen for the study (Annexure 1; Fig. 1). We chose this art form because our objective was to instill concepts of empathy and qualities essential for building a strong doctor-patient relationship. Fine arts, such as painting, evoke emotions, convey stories, and inspire meaning through visual expression.

### Preparation of lesson plan/module and evaluation

With the help of the research team, the lesson plan/module was developed (Annexure 2). The venue, date, and time for the session were fixed, and the roles of the facilitators were clarified. The educational resources for the session were also collated (Annexure 3).

### Internal validation of the lesson plan/module

The research team, i.e., faculty from medical education and ethics, reviewed and validated the lesson plan/ module, and the educational resources.

### Administrative permissions and consents

Appropriate administrative permissions were obtained to conduct the study. The institutional ethics committee approved the conduct of the study [IEC1:80/2023] (Annexure 4). It was clearly communicated to the students that participation was entirely voluntary, and all responses would be anonymized prior to data analysis. Furthermore, it was emphasized that participation in any form would have no impact on their academic progress. They were also informed about their right to withdraw from the study at any stage without any consequences.

## Detailed description of the session

The session based on visual hermeneutics was conducted as a part of the AETCOM module 1.3, “Doctor-Patient relationship” for the students of the first professional year [1]. The timetable has hours mandated for AETCOM and the session on Visual hermeneutics was conducted as a part of the small group discussion (SGD) for the module, “Doctor-Patient relationship.” The session was planned for four groups of students with one facilitator each. Each group consisted of 62–63 students.

### Details of the session (as described in the module/lesson plan, Annexures 2 and 3)

The SGD commenced with brief greetings and an introduction to the module. The conduct of the session followed a three-step approach.

#### Step 1: Introduction to the painting- sir Luke Fildes’ 1891 “The Doctor”

Sir Luke Fildes’ 1891 painting- “The Doctor” [10] was shown to the students. The students were asked to observe the painting keenly. The facilitator posed a question to trigger discussion, “*What do you see*,* what do you feel by looking at****The Doctor****painting*?”

#### Step 2: Self-understanding and interpretation of the painting

Students were encouraged to interpret the artwork based on their understanding. The students updated their thoughts in the form provided (Microsoft Forms) (Annexure 5). The students reflected on the painting with their thoughts and later discussed it with their peers.

#### Step 3: Debriefing and interpretation of the painting by the facilitator

Following it, the facilitator discussed their understanding of the painting, i.e., the use of the image to depict the attributes of a good doctor, the essence of humanity, compassion, and ethics seen in the picture. The facilitator thus attempted to reiterate the importance of the “Doctor-Patient” relationship using the painting. The session was further supported by a short lecture on “Doctor-Patient relationship” followed by discussion covering the following topics: ‘Trust in the doctor-patient relationship’, ‘Rights of a patient and Duties of a doctor’, ‘Boundaries in the doctor-patient relationship’ (Annexure 3).

## Session evaluation

The session’s effectiveness was evaluated based on the first two levels of the Kirkpatrick evaluation model [15, 16]. The first level focuses on recording learners’ reactions and rating their experience, while the second level captures the knowledge and insights participants gained from the session.

### Level 1-reaction

The feedback of students was gathered immediately after the educational intervention. After obtaining informed consent, the students were invited to answer a newly developed and validated semi-structured questionnaire on the session’s effectiveness (Annexure 6).

### Questionnaire validation

The questionnaire was validated for its content by five subject experts (Experts in the field of medical education and bioethics) (Annexure 7), following which the questionnaire was administered to a group of 20 s-year medical students for estimating the reliability of the questionnaire.

After obtaining the informed consent, the questionnaire was administered online via Microsoft Forms. The final study questionnaire consisted of 15 questions designed to evaluate the usefulness of the session. The questions (13 out of 15) were scored based on a 5-point Likert scale from ‘strongly agree’ to ‘strongly disagree.’ The final two questions were open-ended questions about the overall quality of the session and suggestions for further improvement.

### Level 2- learning

The students were asked to present the learnings from the session with reflective writing [17, 18]. Their writings would include the following points.

Description of the session.

What did I learn?

- Self-understanding & reflections on the painting.
- ‘Trust in the doctor-patient relationship’.
- ‘Rights of a patient and Duties of a doctor’.
- ‘Boundaries in the doctor-patient relationship’.

The learnings from this session that I wish to apply in my future role as a healthcare provider or caregiver.

The students uploaded the reflections as an assignment in the learning management system (LMS), i.e., Brightspace-LMS (D2L corporation, Kitchener, Canada) of the authors’ institute for evaluation.

The scores from the assignment contributed to the internal assessment that decides eligibility for first year university examinations. However, this module accounted for only a small percentage of the overall assessment. The students who did not attend this module could still qualify for university examinations.

## Data analysis

### Level 1

The quantitative data was analysed through SPSS 16 (IBM, SPSS Inc). The close-ended questions were evaluated and expressed in percentage. The qualitative data from responses to the open-ended questions was analyzed thematically, and key themes were identified. The authors coded the material after reading and rereading the content. The codes/key words were then examined to find the pattern. To identify the themes, an inductive and semantic technique was used [19].The authors SP and KRN independently identified the codes and arrived at the themes, after which the themes with 90% consensus were retained. The final set of themes underwent rigorous scrutiny, with EK and DAP verifying the themes by comparing them with the original dataset. The descriptions, along with representative quotes fitting each theme, were reviewed by the author UK. To ensure transparency and credibility of the findings, the team engaged in regular self-reflection and discussions throughout the process. This iterative approach enhanced the rigor and reliability of the thematic analysis.

### Level 2

The student entries were evaluated using the rubrics created for reflective writing and graded (maximum score: 10 marks) (Annexure 8). The grades were expressed in the form of mean scores. The specific patterns emerging from the reflective writings were also identified.

## Results

The questionnaire designed to assess the students’ perception of the usefulness of the session was based on the following domains: learner needs, aligning resources for effective teaching, effective facilitation skills, learning climate/environment, time management, and learner satisfaction.

### Questionnaire validity and reliability

The Content Validity Ratio (CVR) was calculated for each item, and the Content Validity Index (CVI) was calculated for the overall instrument. The CVI for the questionnaire’s relevance, clarity, and essentiality were 0.947, 0.707, and 0.973, respectively. The low score in the CVI of ‘clarity’ was corrected by incorporating the necessary changes/ suggestions and received complete agreement. Based on the reviewer’s recommendations and necessary incorporations, the final questionnaire consisted of 15 items, with the last two being open-ended. The reliability of the questionnaire was confirmed with Cronbach’s alpha of 0.91 ensuring reliability.

### Session findings

**Fig. 2:**
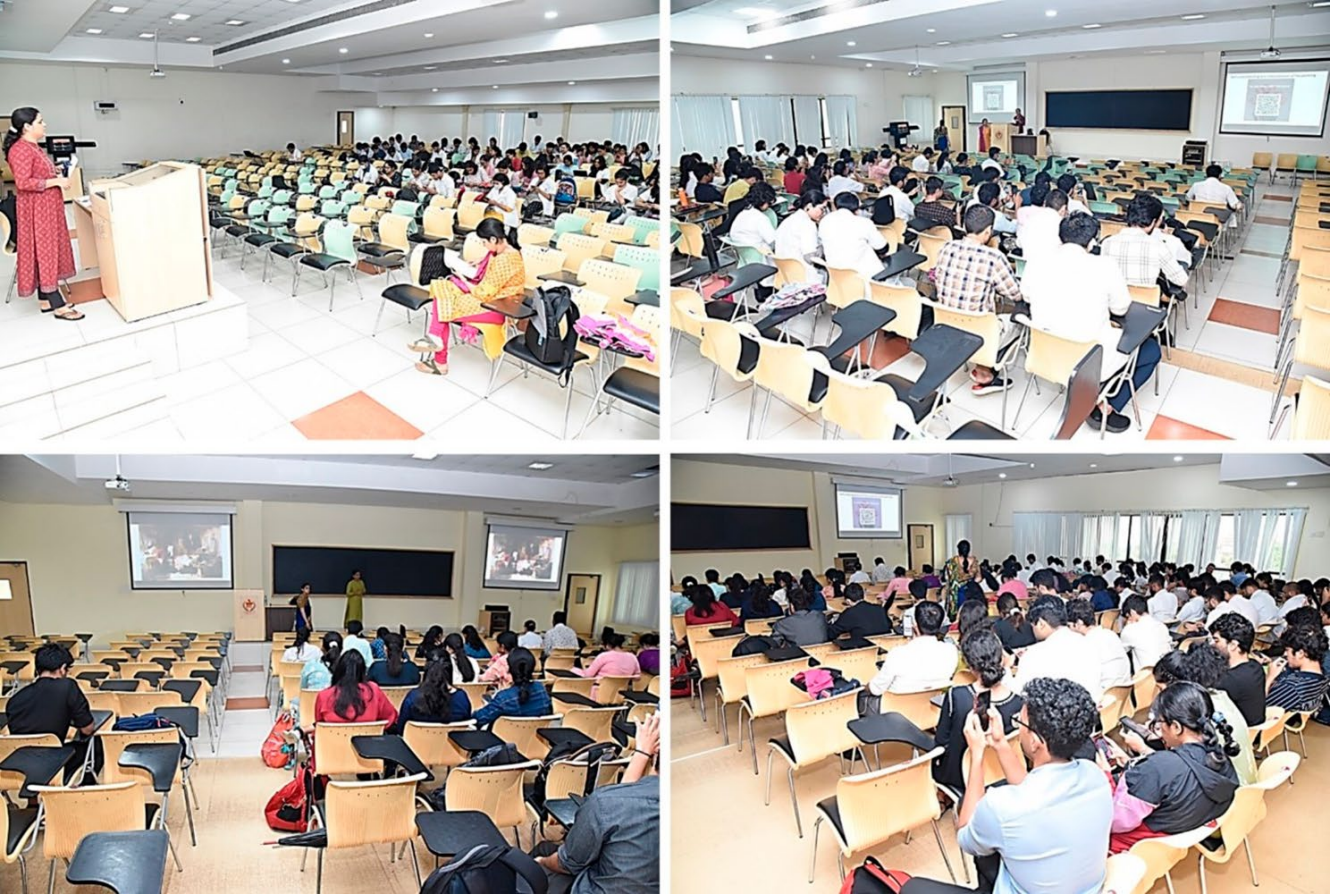
Excerpts from the session conducted as a part of the AETCOM Module: “Doctor-Patient Relationship”

One hundred and sixty students (74 males and 85 females) consented to be part of the study. Due to the space constraints that arose due to unavoidable and restricted logistics, the students were divided into two groups of 80 students each. They were housed in two separate lecture halls. The session was conducted in parallel by two facilitators in each lecture hall (Fig. 2).

**Fig. 3:**
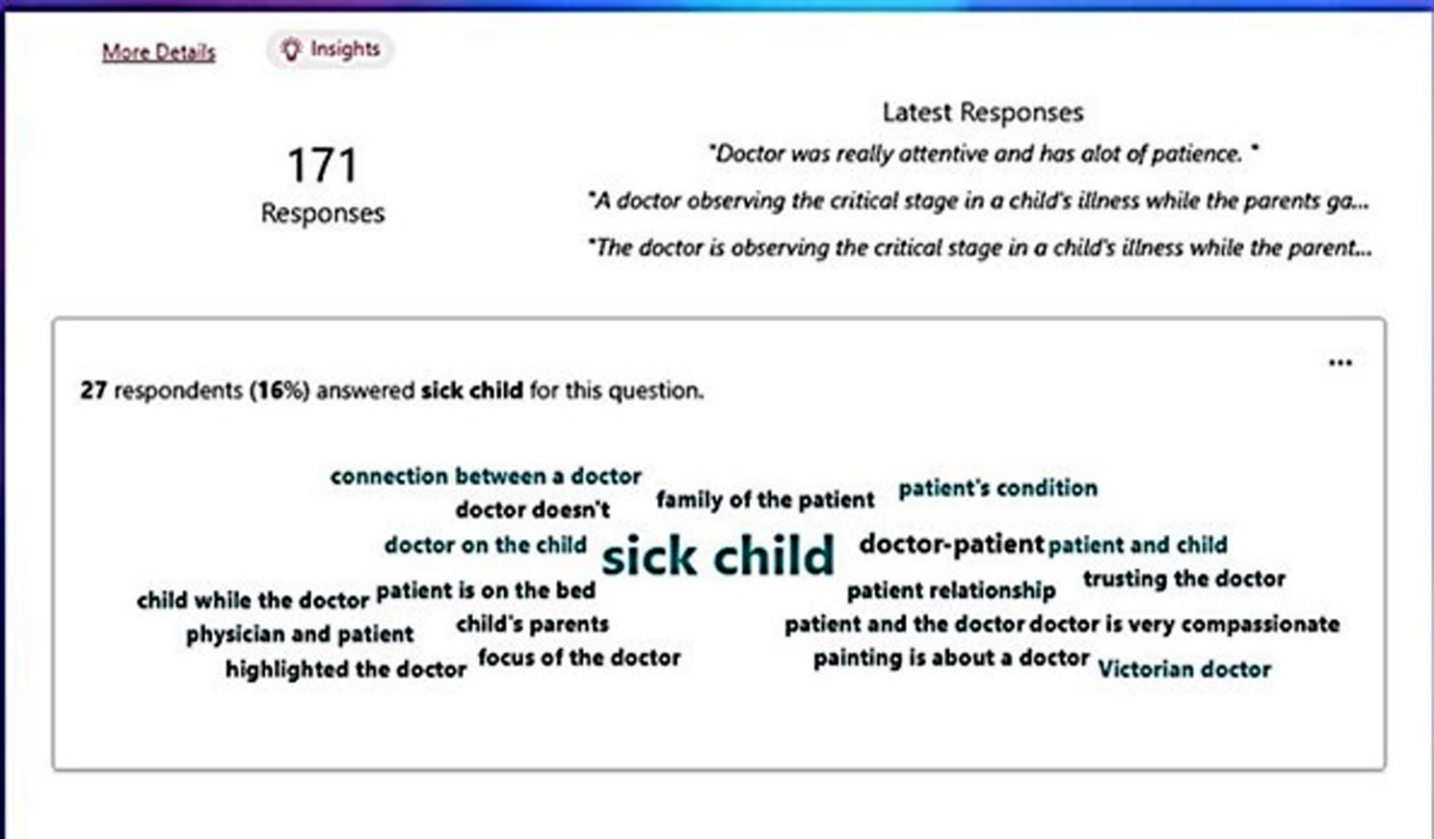
Pictorial representation (Screenshot) of the keyed words provided as responses to the Self-understanding and interpretation of the painting

The age of the participants ranged between 18 and 23 years (mean age: 19).

### Findings: self-understanding and interpretation of the painting

The students understood the image with mere observations, although significant details had to be addressed in subsequent discussions. Analysis of the responses to the students’ self-understanding and interpretation of the painting revealed the ‘Doctor-patient relationship’ as the core theme. The keywords identified were: ‘sick child,’ ‘focus on the doctor,’ ‘anxious parents’, ‘trust in the doctor,’ ‘doctor and patient’, and ‘compassionate doctor.’ (Fig. 3, Supplemental data 1). The learners could decipher that a doctor who looks very compassionate and caring is taking care of a sick child in a critical condition with anxious parents, mother being worried while father awaits with courage and patience.

It shows a doctor trying to treat a little kid. The parents of the kid can be seen in the background, the mother is crying and praying for her child to be cured, the father is trying to gather all his courage and patience, his pain can still be felt (Learner 1).

It is about a child being in critical condition, there is a doctor seeing the patient and child’s parents are in a lot of worry, her mother is praying, pleading please save her (Learner 2).

It depicts a compassionate and dedicated doctor sitting by the bedside of a sick child while the child’s worried parents watch attentively. The painting captures the essence of the doctor’s caring nature, and the Trust placed in him by the family during a time of illness. It remains an iconic representation of the selflessness and dedication of medical professionals in their pursuit of healing and helping others (Learner 3).

## Session evaluation

### Level 1-REACTION: learner satisfaction or reaction to the program

**Fig. 4:**
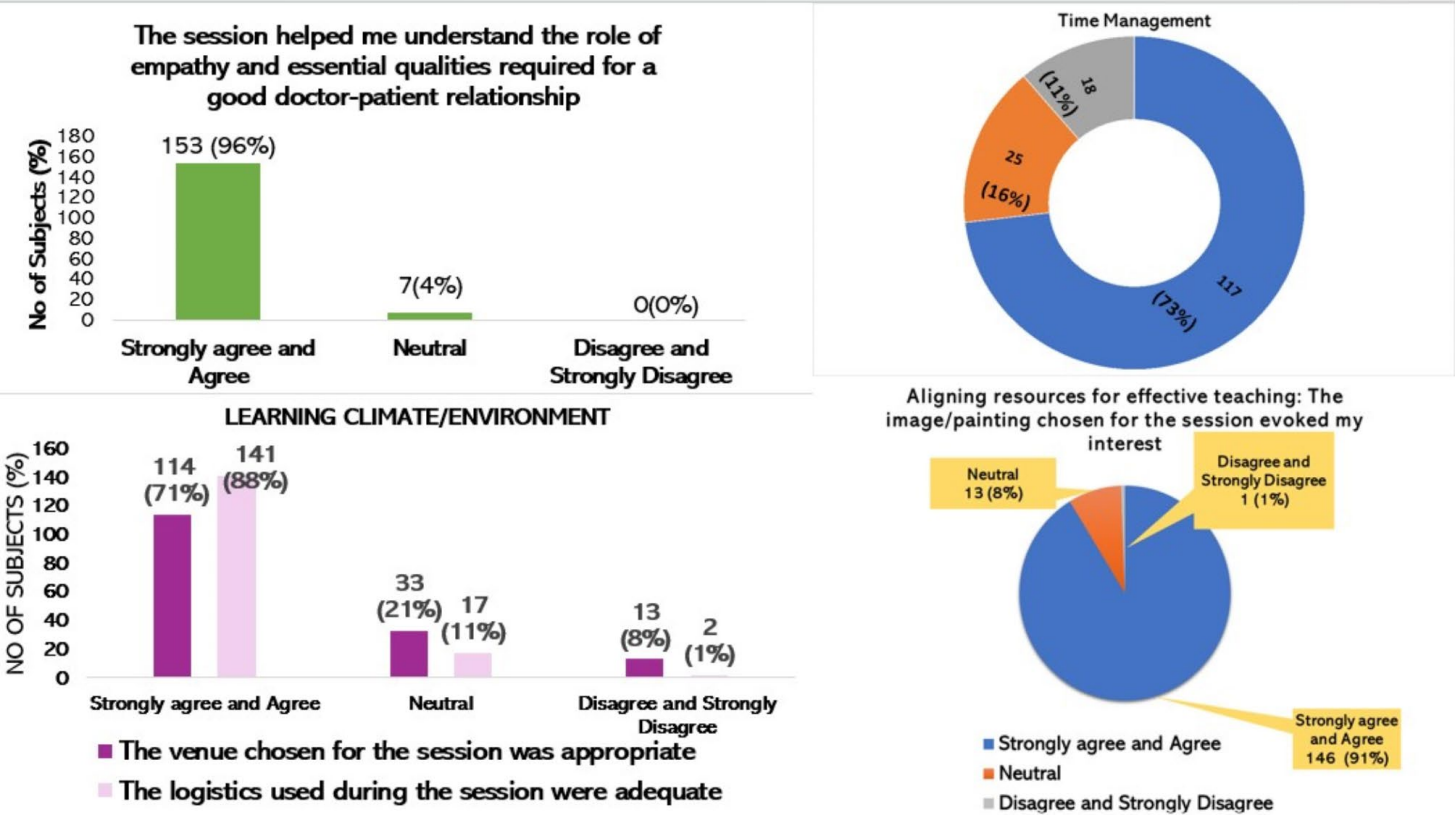
Graphical representation of the students’ perception about the session usefulness related to ‘learner needs,’ ‘aligning resources for effective teaching,’ ‘learning climate/environment,’ and ‘time management.’

**Fig. 5:**
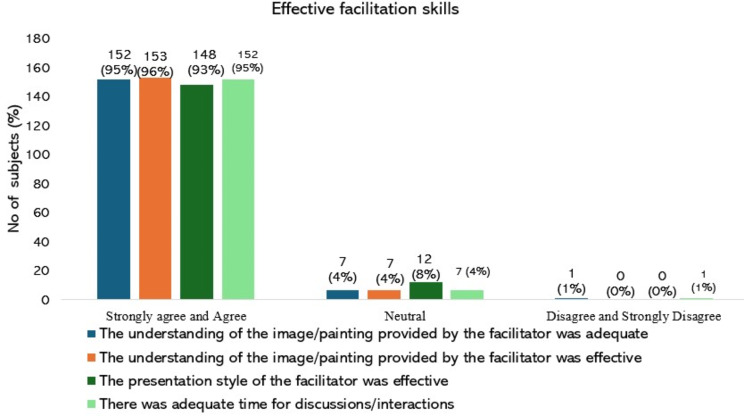
Graphical representation of the students’ perception about the session usefulness related to ‘Effective facilitation skills.’

**Fig. 6:**
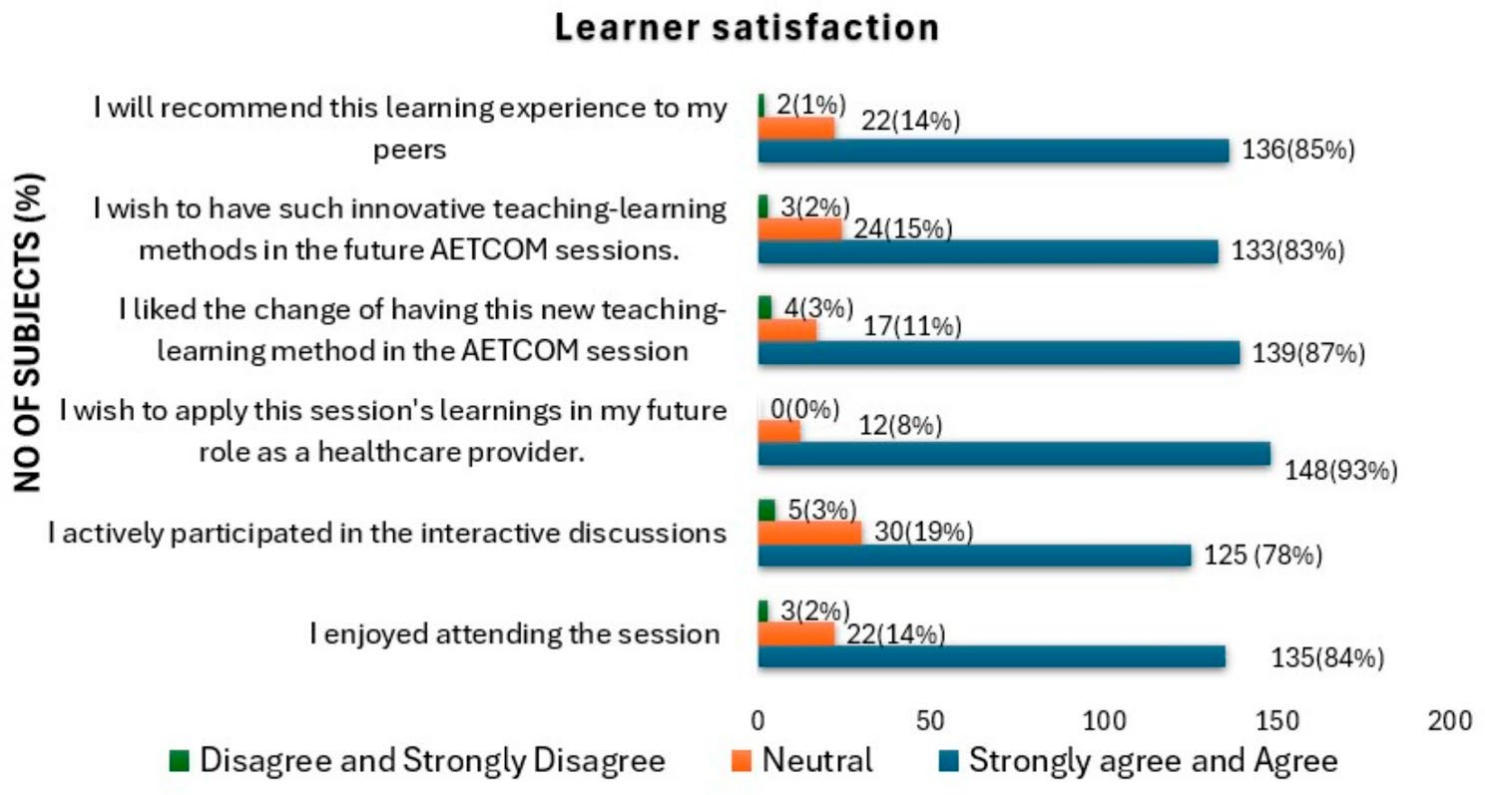
Graphical representation of the students’ perception about the session usefulness related to ‘learner satisfaction.’

Students’ perception of session usefulness is graphically represented in Figs. 4, 5, 6. (Supplemental data 2). Most of the students (96%) were happy with the conduct of the session and agreed that it helped them understand the qualities required for a good doctor-patient relationship. Majority of the students (91%) affirmed that the image/painting chosen for the session evoked their interest. Nearly 95% stated that the facilitator provided an adequate understanding of the image/painting. It was also observed that the presentation style of the facilitator was effective (93%), and there was sufficient time for discussions/interactions (95%). The opinion on whether the venue chosen for the session was appropriate found varied responses, with nearly 71% agreeing, with 21% being inconclusive. The logistics used during the session were adequate, as stated by 88% of the participants. The duration of the entire session was adequate, as agreed by 73% of the students. While 84% of the participants enjoyed attending the session, 78% said they actively participated in the interactive discussions. Most participants (93%) agreed that they would apply the session’s learnings in their future role as a healthcare provider. Participants (87%) appreciated the introduction of this new teaching-learning method in the AETCOM session and expressed a desire for more innovative approaches in future sessions (83%). The average rating of the overall learning experience put forth by the participants (One being ‘worst’ and ten being ‘best’) is 8.63.

**Fig. 7:**
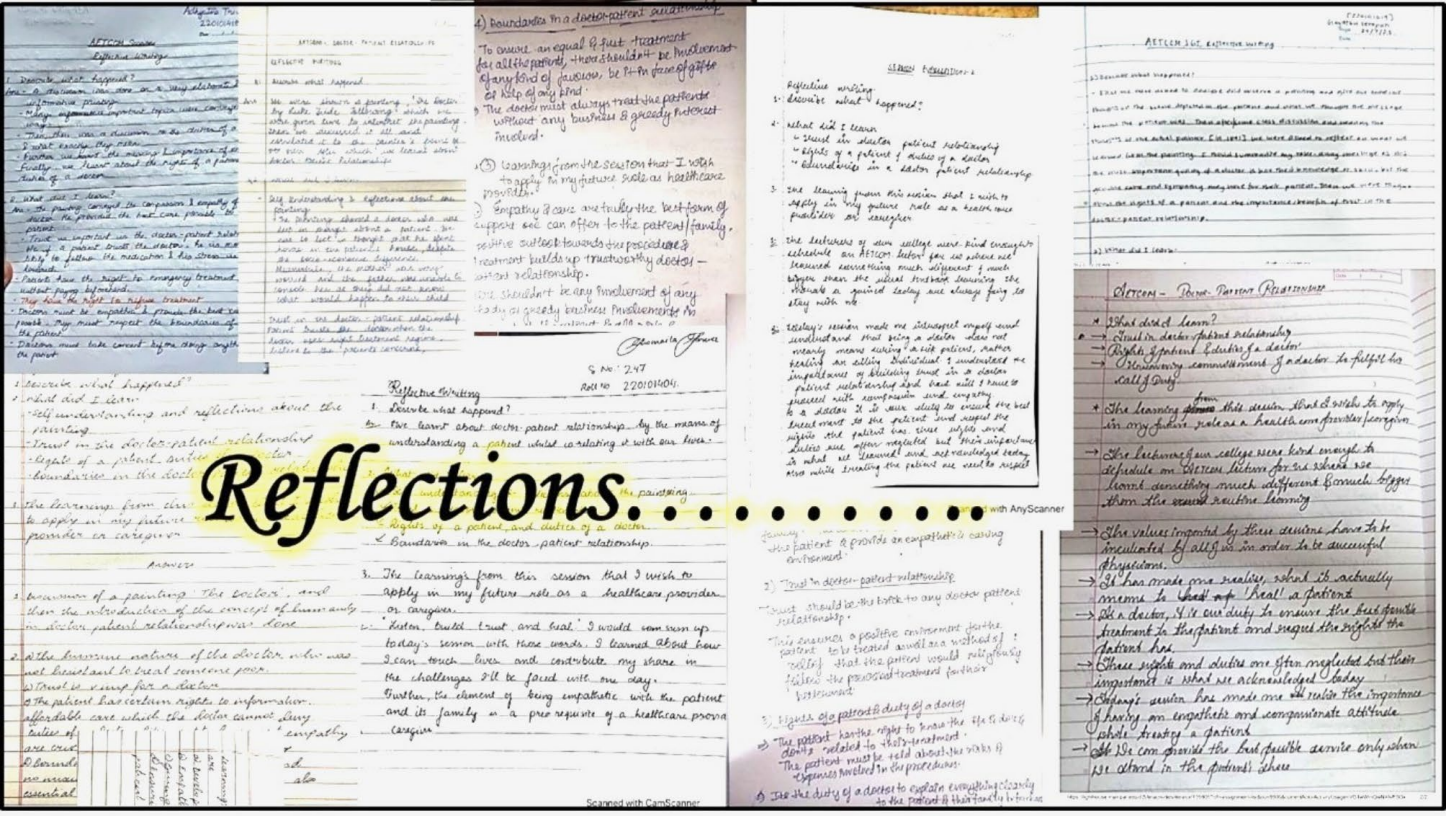
Representative images of a few ‘reflective writing’ entries

The responses to the open-ended questions were analysed thematically. An inductive approach was followed, and the following themes were derived:

#### Theme 1. Fostering insightful learning through interactive discussions

The analysis of the open ended question “What did I like about the session?” from the questionnaire revealed key words such as *Painting teaching-“Poochta Pictures”!!; Interactive session*,* Thought-provoking discussions*,* Insightful learning*,* Emotional painting*,* Interesting session.*

The participants liked the session entirely. They took the idea of using paintings to learn empathy in doctor-patient relationship. They added that the process was highly interactive, and the painting provided greater learning exposure. It was interesting to note that one of the students coined the word “*Poochta pictures*” (English translation for Hindi: Asking pictures), stating that the painting asked questions and made them think about the importance of doctor-patient relationship. It was an emotional experience that led to thoughtful discussions and insightful learning.

The explanations and the method of teaching were excellent, and the picture activity got my interest (Learner 1).

I liked the fact that we were shown a painting, and we were told to reflect on our thoughts regarding the painting. We were not taught straight from a PowerPoint but were interacting and learning about the doctor-patient relationships on our own, with help from the teacher. (Learner 2).

The way we were taught doctor patient relationship was very innovative and interactive, I liked the way the teacher took an image and explained the very basis of the Poochta Pictures (Learner 3).

I think the story behind the painting was really very emotional, and it totally drew me towards it; it was a really beautiful story, and I loved it! (Learner 4).

To allow us to think about the painting before explaining its true meaning, which told us how different scenes play a role (Learner 5).

I like the new way of teaching us the doctor-patient relationship through visuals, that is, through pictures. (Learner 6).

#### Theme 2. Enhancing realism through multiple paintings

While participants appreciated the overall conduct of the session, they provided few suggestions for further improvement. They recommended multiple paintings to be incorporated to enrich the session and emphasized the need for the depiction of real-life situations and scenarios. The learning environment could have been more congenial, as suggested by some of the participants. They mentioned that it would be beneficial to have smaller groups that would have enhanced the discussions and learning. They felt the lecture part of the session could be shortened to involve more discussions.

Inclusion of more paintings in this kind of sessions (Learner 1).

I felt the lecture part of the session was a little long and maybe a little monotone (Learner 2).

More pictures could be shown (Learner 3).

Dividing the class into even smaller groups and using stories rather than paintings (Learner 4).

More real-life examples of incidents including doctors performing their roles, and situations in which they faced problems. Learning how to handle such situations would be beneficial (Learner 5).

### Level 2-LEARNING

#### Students’ learnings from the session evaluating its usefulness

The participants’ reflections presented the learnings from the session well. The importance of Trust in the doctor-patient relationship, the patient’s rights, the doctor’s duties, and the boundaries in the doctor-patient relationship were also beautifully detailed by most participants. The reflective writings submitted by the participants were evaluated using the rubrics. A mean score of 7.906 (maximum score: 9.5 and minimum: 5 out of 10) was obtained.

The specific patterns observed in the reflective writings are described below:

##### Emphasis on Empathy and Compassion

Learners consistently highlighted the importance of stepping into the patient’s shoes, understanding their emotions, and treating them with kindness and respect.

Today’s session has made me realize the importance of having an empathetic and compassionate attitude while treating a patient. We can provide the best possible service only when we stand in the patient’s shoes. (Learner 1)

I will try to have empathy for all patients; I will respect personal boundaries (Learner 10).

##### Trust in Doctor-Patient Relationship

Building and maintaining trust was emphasized as vital for effective healthcare delivery. Trust was linked to patient comfort, transparency, and adherence to treatment plans.

I learned that in a doctor-patient relationship, having Trust in one another and communicating the problems properly plays a great role. It is also to be kept in mind that it becomes doctor’s responsibility when their patient shows Trust in them to not only treat the patient but also understand their condition and circumstances (financially, emotionally, physically & mentally). (Learner 2)

I want to become a doctor who patients can trust easily and share their problems without any discomfort. (Learner 4)

Developing Trust in patient so that he will follow up on your medication and come again to you. (Learner 5)

##### Communication skills is essential in building trust

Learners emphasized the need for clear, jargon-free communication and attentiveness to patients’ perspectives. Effective communication builds trust and ensures patients understand their care plans.

Always maintain a good relationship with the patient. Communicate properly. Do not use medical terminology in front of the patient and scare him out. The duties of doctor which we must do when we serve as healthcare provider. Respect the patient deny for treatment. (Learner 5)

“Listen, build, trust and heal.” I would sum up today’s session with these words. I learned about how I can touch lives. (Learner 8)

##### Ethical and Professional Conduct

Ethical decision-making, avoiding exploitation, and prioritizing patient welfare over financial gains were the recurring ideas observed in the reflections.

Empathy and care are truly the best form of support one can offer to the patient/family. A positive outlook towards the procedure and the treatment builds up a trustworthy doctor-patient relationship. There shouldn’t be any involvement of any shady or greedy business involvements in the treatment of the patient, and all risks and expenses must be mentioned to the patient and family beforehand. (Learner 6)

The learning from this session that I wish to apply in the future is giving my undivided attention to the patient, I am attending at that moment; not considering financial benefits & valuing patient’s interest (Learner 3).

##### Lifelong learning and Leadership

Learners expressed a desire to continuously expand their knowledge and skills to provide better care.

A doctor should be a good healer, compassionate, good planner, health advocate. (Learner 7)

I will learn to use my knowledge & skill set & to keep expanding my knowledge to provide patients with the best possible healthcare. (Learner 9)

I will consider it my responsibility to educate the public about health and medicine. (Learner 10)

Representative images of a few reflections are depicted in Fig. 7. A few notable reflections are also included in the annexures (Annexure 9).

## Discussion

To build a meaningful doctor-patient relationship during the initial years of medical training, appropriate measures need to be taken to instil and improve the quality such as empathy among learners.

Introducing empathy, and qualities for building a good doctor-patient relationship into the early years of undergraduate medical education is crucial for producing well-rounded and compassionate healthcare professionals [20]. This approach introduces novice learners a comprehensive understanding of their profession, including its challenges, responsibilities, and privileges [1].

Before the NMC-mandated AETCOM course in the curriculum, the doctor-patient relationship was meant to be imbibed by the learners, i.e., the medical students, by mere observations. The students learned the basics of the doctor-patient relationship by shadowing or observing the doctor, i.e., a senior physician or practitioner seen interacting with patients [21]. Senior peers also assisted the juniors in the learning process. However, with the increased burden of managing high patient numbers and reliance on investigation modalities for patient diagnosis, the casual communication between a doctor and patient grew shorter and the discussions centred around prioritizing disease diagnosis over actively listening to the patients and family [22]. It was observed that most students were getting predominantly trained in the disease-centred rather than in the patient-centric approach [23].

To strengthen attitude, ethics and communication skills, the NMC mandated the introduction of the AETCOM course in the undergraduate medical curriculum of Indian schools [24] and we found a window of opportunity to introduce attributes of a good physician and empathy concepts using visual hermeneutics in the AETCOM module on the doctor-patient relationship in the first professional year.

Doctors are expected to be trustworthy, moral, honest, compassionate, and accountable working in the best interests of the patient. Trust, a most important quality in doctor-patient relationship gets developed when doctor shows genuine interest in their patients and sensitivity to their emotions [25] The students in our study expressed an understanding of the trust that patients and their families place in the treating physician and how the physician, in turn, reciprocates by prioritizing the patients’ well-being.

Visual arts can foster reflective thinking capacity and are an innovative and valuable educational tool. Hermeneutics is the discovery of creation and meaning and drawing on the power of observation, visual hermeneutics is a novel method to cultivate interpretative and reflective thinking skills [7]. In our study, Sir Luke’s paintings provided learners with an opportunity to explore underlying stories, meanings, and emotions entwined in the doctor-patient relationship while also reflecting on their own emotional responses. Learners described the experience as deeply emotional, leading to thoughtful discussions that deepened their understanding of empathy and its role in clinical practice.

Visual Thinking Strategies (VTS), like visual hermeneutics, emphasize interpreting visual materials through observation, critical thinking, and collaborative discussion to enhance visual literacy, the ability to comprehend information conveyed in pictorial or graphic forms. Visual hermeneutics further emphasizes contextual and interpretative analysis to uncover deeper meanings and narratives within visuals [26]. Similar to our study, Bentwich et al. employed a VTS-based approach to teach visual arts, demonstrating its potential not only to foster empathy but also to help preclinical medical students develop tolerance for ambiguity, a critical skill in clinical practice. However, our methodology diverged by engaging learners more deeply with the interplay of emotions and stories within the doctor-patient relationship [27].

Earlier, Gurwin et al. introduced art observation training for first-year medical students, employing the ‘artful thinking’ approach during live museum sessions. While artful thinking shares similarities with visual hermeneutics, both engaging learners with visual stimuli, artful thinking distinctly focuses on thinking dispositions and thinking routines such as reasoning, questioning, observing, describing, comparing, and connecting to encourage the generation of diverse viewpoints [28]. Visual arts such as integrative art-museum programs can facilitate clinically relevant learning across various competencies, including doctor-patient relationship [26, 29]. It can broaden students’ understanding of patient encounters. Visual arts were used to promote active, deliberate observation of emotions within artwork and awareness of one’s own emotional responses. Similar to our study, Rezaei S et al. introduced concepts of bias and empathy to first-year medical students through visual observation skills, incorporating drawing and art interpretation exercises [9].

While we have used hermeneutics to teach concepts of empathy and the doctor-patient relationship through visual arts, the application of the hermeneutic window has been explored within clinical education. This approach encourages trainees to critically examine their values, beliefs, biases, and assumptions, helping them overcome barriers to effectively communication with patients during consultations [30]. This approach can enable trainees to more fully engage with the complexities and uncertainties inherent in patient care, fostering greater learner engagement and enhancing professional fulfilment.

Molding the learners in early phase is beneficial, and previous studies have focused on leveraging the capabilities of visual arts to foster non-technical skills among preclinical medical students. In addition to the acquisition of observational skills and empathy, integration of visual arts into the curriculum through pedagogical approaches such as structured art museum tours, focused observational sketches, verbal imaging, journalling and reflections has been shown to help students to develop team building, communication skills, resilience and cultural sensitivity [10]. He B et al. performed a qualitative analysis of reflections after curated art gallery visits. In addition to students developing observational skills, they found that students developed personal and professional identities, ethical values and professionalism expected of a physician [31]. Protected time with artwork stimulated personal reflection and promoted sense of enrichment and wellbeing. An interdisciplinary workshop approach using portraits, paintings, and racially charged content has shown to help students critically reflect, learn empathy, understand cultural sensitivity and address racial issues, ultimately enhancing their ability to manage concerns related to vulnerable patients [32, 33].

Developing strong observational skills and teaching learners to articulate their observations with attention to detail, enables them to pick up on subtle cues during patient interactions, ultimately supporting more accurate clinical diagnoses. With the advent of artificial intelligence (AI) in medical education, AI generated art can further enrich learner experience by creating visual representations of patient experiences or healthcare provider perspectives that might be difficult to express in words alone [34].

Analysis of the usefulness of the teaching module in the present study revealed that the students took the idea of using paintings to understand the roles and boundaries to be exercised in doctor-patient relationship. They added that the process was highly interactive, and the painting provided greater learning exposure. The painting asked questions and made them consider the importance of doctor-patient relationship. It was an emotional experience that led to thoughtful discussions and insightful learning. The students in our study were engaged in reflective writing assignments. Writing reflections allows learners to process and express their emotional responses to the art, helping them connect with the emotional experiences portrayed. This process builds empathy, which is essential in the patient care [35]. Also, reflective writing allows learners to deepen their understanding and internalise the experience as described by the Gibbs reflective writing cycle framework. Reflective practice encourages learners to become aware of one’s own bias and attitude and how their perceptions can influence communication with patients [17, 36].

## Limitations and future recommendations of the study

### Limitations

Although the study was originally designed to be conducted in small groups, unforeseen constraints such as the unavailability of demonstration rooms during the session necessitated its implementation in larger groups. Efforts were made to engage learners and maintain interactivity in the large group format. However, it was challenging to hear responses from all students during the discussions, limiting comprehensive participation. We understand that the discussions would have been more enriching in smaller groups, where facilitators can ensure and encourage active participation.

We encouraged students to submit reflective writing assignments within a week after the in-class activity to avoid recall bias that would have potentially distorted their initial perceptions. During thematic analysis, we made attempts to connect to broader understanding of doctor-patient relationship rather than narrow constructs of meaning from the painting.

While our study guided learners through reflective thinking-based assignments, it is limited by the fact that the hermeneutic approach was introduced in a single teaching-learning session.

We did not include a control group in the study. As we were implementing a new teaching approach, managing a large cohort of students within the limited time slots available in the annual schedule posed a significant challenge. Also, balancing multiple course requirements made it difficult to design a study with two distinct teaching approaches while ensuring an equitable experience for both the case and control groups. Future studies can aim at incorporating a control group in the study design.

### Recommendations

Future studies could explore longitudinal study designs that integrate hermeneutics through visual arts in multiple AETCOM modules as students advance over professional years. Future research in AETCOM could examine the use of diverse visual materials such as photography, graphic narratives, infographics, sculptures, cartoons, and comics. These materials can help educators tailor their teaching to specific topics, that could possibly cover areas like communication skills, socio-cultural and economic aspects, and medico-legal issues.

In addition to these explorations, it is crucial to consider the potential for scaling these modules across different cultural and institutional contexts. We recommend the adoption of these strategies and extend to all health professional institutions, including medical, dental, nursing, and allied health schools, forming interprofessional teams of learners to think in ways to bridge professional boundaries and enhance mutual understanding. By implementing these modules across various health disciplines, the training can aim at creating awareness of cultural and professional diversity, reflective practice to overcome biases and values that influences patient care while working in teams. Feedback from pilot studies conducted in diverse educational settings could provide valuable insights into the adaptability and effectiveness of novel pedagogic techniques. By identifying cultural and institutional requirements, educators can customize materials and methods to suit specific learner needs.

The incorporation of digital and AI tools, such as AI-generated art forms and immersive virtual reality, can further enhance the scalability and adaptability of visual hermeneutics in AETCOM modules. These technologies can help address contextual challenges by fostering creativity, personalization, and deeper learner engagement, while ensuring inclusivity and relevance across a broad range of learning environments. Also, adoption of interactive digital platforms can allow collaborative annotation and interpretation of artworks in real time. Each participant can contribute their insights, fostering engagement in a large cohort of learners.

## Conclusion

The present study constructed a teaching module for introducing attributes of good physician and empathy in doctor-patient encounters in the AETCOM module on the “Doctor-patient relationship” for first-year medical students using paintings and visual hermeneutics.

The practice of visual hermeneutics can help cultivate the virtues of empathy and kindness in students during their early years of undergraduate medical training. The learners expressed satisfaction with the teaching module and recognized the importance of empathy and the essential qualities required for building strong doctor-patient relationship. This understanding is essential for developing competent healthcare professionals who not only possess medical expertise but also approach patients with care and a genuine desire to promote their well-being.

In summary, incorporating visual hermeneutics into the “Doctor-patient relationship” module for first-year medical training encourages students to interpret and appreciate the human aspects of healthcare, nurturing empathy and goodness, possibly preparing them to be more effective and compassionate healthcare providers in their future careers. Visual hermeneutics, being a promising approach, can be incorporated into other AETCOM modules to teach concepts such as medico-legal, socio-cultural, and ethical principles related to patient care.

## Electronic supplementary material

Below is the link to the electronic supplementary material.

Supplementary Material 1

Supplementary Material 2

Supplementary Material 3

Supplementary Material 4

Supplementary Material 5

Supplementary Material 6

Supplementary Material 7

Supplementary Material 8

Supplementary Material 9

## Data Availability

The datasets generated and/or analysed during the current study are available in the [FIGSHARE] repository, [https://figshare.com/s/cba4e80cd45f3ff36eea] [37].

## Abbreviations

AETCOM: Attitude, Ethics, and Communication
UGs: Undergraduates
NMC: National Medical Commission
MCI: Medical Council of India
GMER: Graduate Medical Education Regulations
SGD: Small group discussion
LMS: Learning management system
CVR: Content Validity Ratio
CVI: Content Validity Index
AI: Artificial intelligence
