# Supplementary figures and images for: Visual hermeneutics as a tool to introduce empathy and core physician attributes in doctor-patient relationship for first-year medical undergraduate students
Source: BMC Med Educ. 2025 Jan 29;25:145. doi: 10.1186/s12909-025-06742-6 (PMC11780788; doi:10.1186/s12909-025-06742-6)

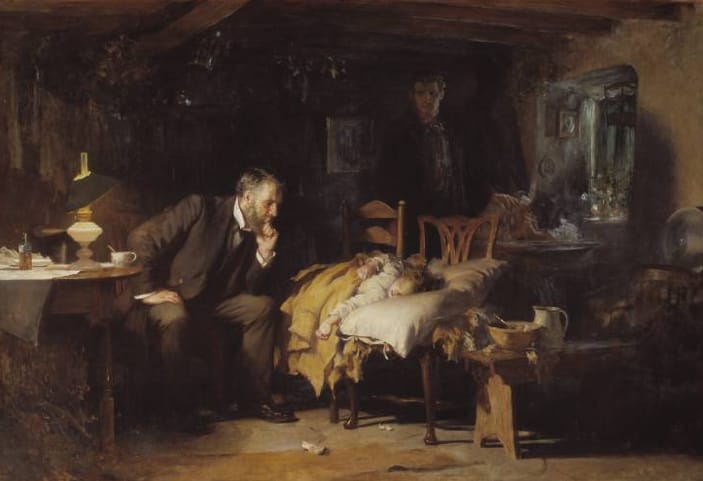

Supplement: Supplementary file 1 — Supplementary Material 1 [file 12909_2025_6742_MOESM1_ESM.jpg]
