# Supplementary material for: Visual hermeneutics as a tool to introduce empathy and core physician attributes in doctor-patient relationship for first-year medical undergraduate students
Source: BMC Med Educ. 2025 Jan 29;25:145. doi: 10.1186/s12909-025-06742-6 (PMC11780788; doi:10.1186/s12909-025-06742-6)
Supplement: Supplementary file 2 — Supplementary Material 2 [file 12909_2025_6742_MOESM2_ESM.pdf]

Department of Medical Education
AETCOM Module 3 – Doctor-Patient Relationship

Session plan: The module will be conducted during the small group discussion (SGD) session for the AETCOM module., "Doctor-Patient relationship."
The students will be divided into four batches of 62-63. Each batch will have one facilitator.

Venue: Interact GA, GB, GC, and GD
Date: 29th July 2023
Time: 2-5 pm
Facilitators: Dr. SP, Dr. KRN, Dr. EK, Dr. DAP, Kasturba Medical College Manipal
T-L methods: PowerPoint presentation & Small Group Discussions

Lesson Plan

**Time (PM)**	**Activity**
2.00-2.15	Attendance & Introduction to AETCOM module 3: “Doctor-patient relationship”
2.15-2.30	**Introduction to the painting- Sir Luke Fildes' 1887 “The Doctor”**<br>The students will be shown Sir Luke Fildes' 1887 painting- “The Doctor”.<br>The students will be made to observe the painting keenly and jot down the observation points.
2.30-2.45	**Self-understanding and interpretation of the painting**<br>Students will be encouraged to interpret the artwork based on their understanding. The students will update their thoughts and understanding in the link provided.<br>Students will be invited to discuss their thoughts with the rest of the audience.<br><br>**Facilitator's role** will be to pose a trigger to initiate discussion. “What do you see, what do you feel by looking at *The Doctor* painting”.
2.45-3.45	**Debriefing and interpretation of the painting by the facilitator**<br>The facilitator will discuss their understanding of the painting, i.e., the use of the image to portray the qualities of a good doctor, the essence of humanity, compassion, and ethics seen in the picture.<br>The facilitator will thus attempt to reiterate the importance of the "Doctor-Patient" relationship using the painting.<br><br>***Discussion points (USE PPT):***<br>Professional qualities and roles of a physician<br>Empathy and ethics in patient encounters<br>Duties of doctors<br>Rights and Responsibilities of patients<br>Trust in the doctor-patient relationship<br>Boundaries of the doctor-patient relationship<br><br>**Facilitator's role** will be to pose trigger to initiate discussion.
3.45-4.00	**BREAK - Q&A**
4.00-5.00	**Session evaluation**

<table>
<tr>
<td></td>
<td>

Level 1: The students will be invited to answer a semi-structured questionnaire on the session's effectiveness. The study questionnaire comprises 20 questions that address the usefulness of the session. The questions (17 out of 20) will be scored based on a 5-point Likert scale from 'strongly agree' to 'strongly disagree.' At the end of the questionnaire, there are two open-ended questions about the overall quality of the session, the overall satisfaction of participants, and the learnings derived from the session.

Facilitators role: Display the QR code for the questionnaire and enable students to answer.

Level 2: Reflective writing: The students will be asked to present the learnings from the session**.** Their writings will be assessed for the following: Self-understanding & reflections about the painting, Learnings, and key takeaways from the session. The reflections will be uploaded by the students in the learning management system (LMS), i.e., Brightspace-LMS

Facilitators role: Guide students with the process of reflective writing by stressing on the following points:

- Self-understanding & reflections about the painting
- Learnings, and key takeaways from the session

<u>Reflective writing points:</u>

1. Describe what happened?

2. What did I learn:
-Self-understanding & reflections about the painting
- Empathy and ethics in patient encounters
-'Trust in the doctor-patient relationship'
-'Rights of a patient and, Duties of a doctor
-'Boundaries in the doctor-patient relationship'

3. The learnings from this session that I wish to apply in my future role as a healthcare provider or caregiver

Ask the students to upload the reflections in the learning management system (LMS), i.e., Brightspace-LMS for further evaluation

</td>
</tr>
</table>

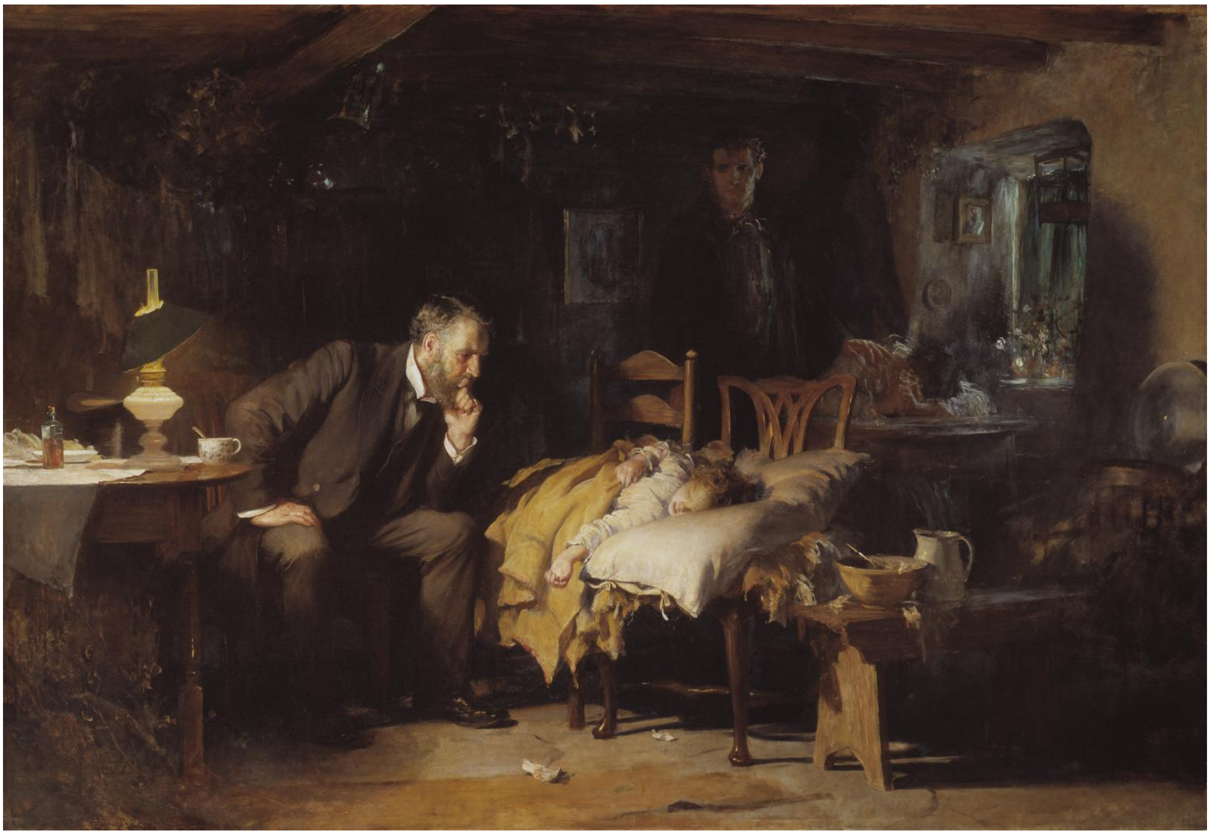
Sir Luke Fildes' 1891 painting *The Doctor*

Further reading:

NMC AETCOM: https://www.nmc.org.in/wp-content/uploads/2020/01/AETCOM_book.pdf
