# Supplementary material for: Visual hermeneutics as a tool to introduce empathy and core physician attributes in doctor-patient relationship for first-year medical undergraduate students
Source: BMC Med Educ. 2025 Jan 29;25:145. doi: 10.1186/s12909-025-06742-6 (PMC11780788; doi:10.1186/s12909-025-06742-6)
Supplement: Supplementary file 3 — Supplementary Material 3 [file 12909_2025_6742_MOESM3_ESM.pdf]

AETCOM Module 3
“Doctor Patient Relationship”

LEARNING OBJECTIVES

By the end of the session, one must be able to:

- Understand & reflect on the painting- “The Doctor”
- Explain the features of ‘Trust in the doctor-patient relationship’
- Understand the Rights of a patient and, Duties of a doctor
- Understand the boundaries in the doctor-patient relationship’

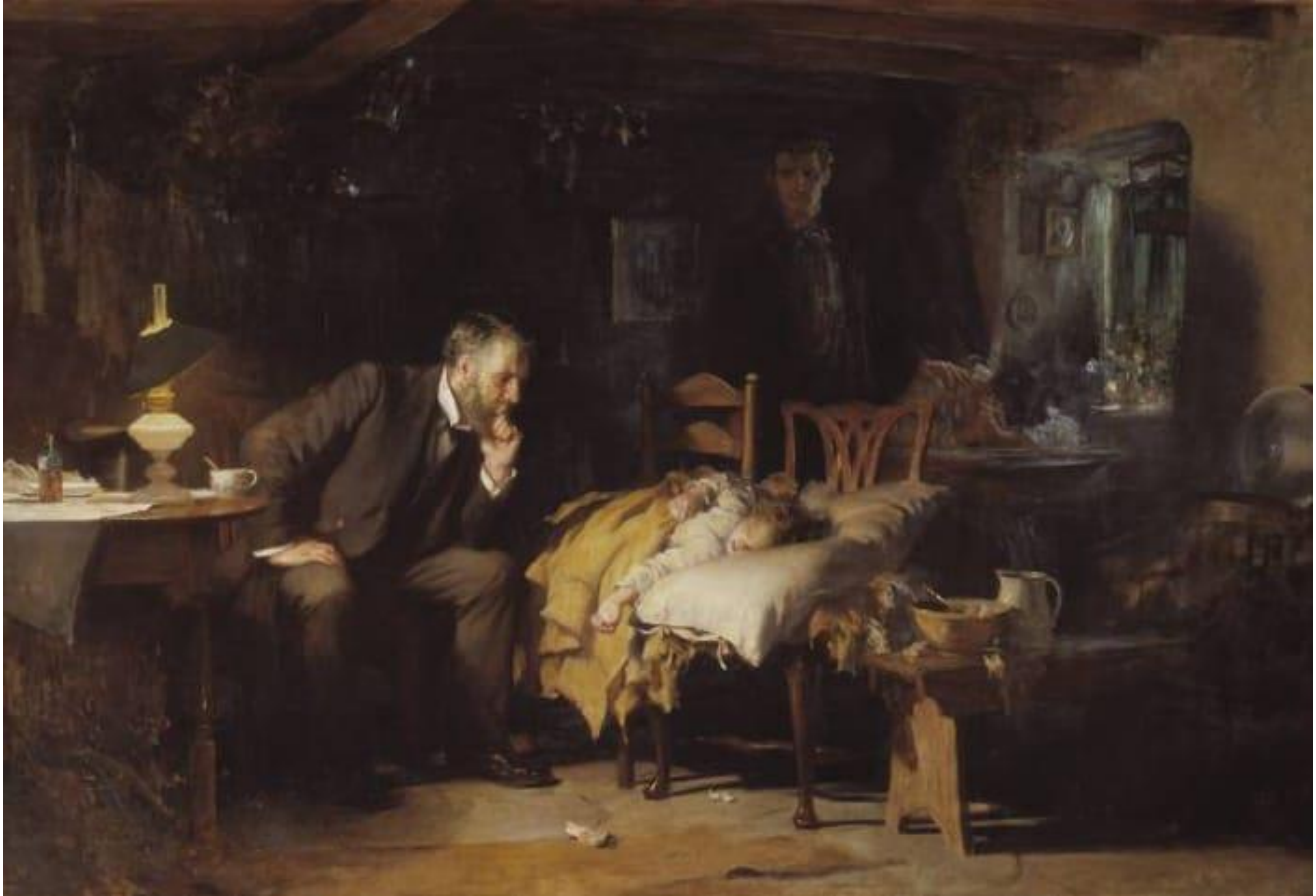

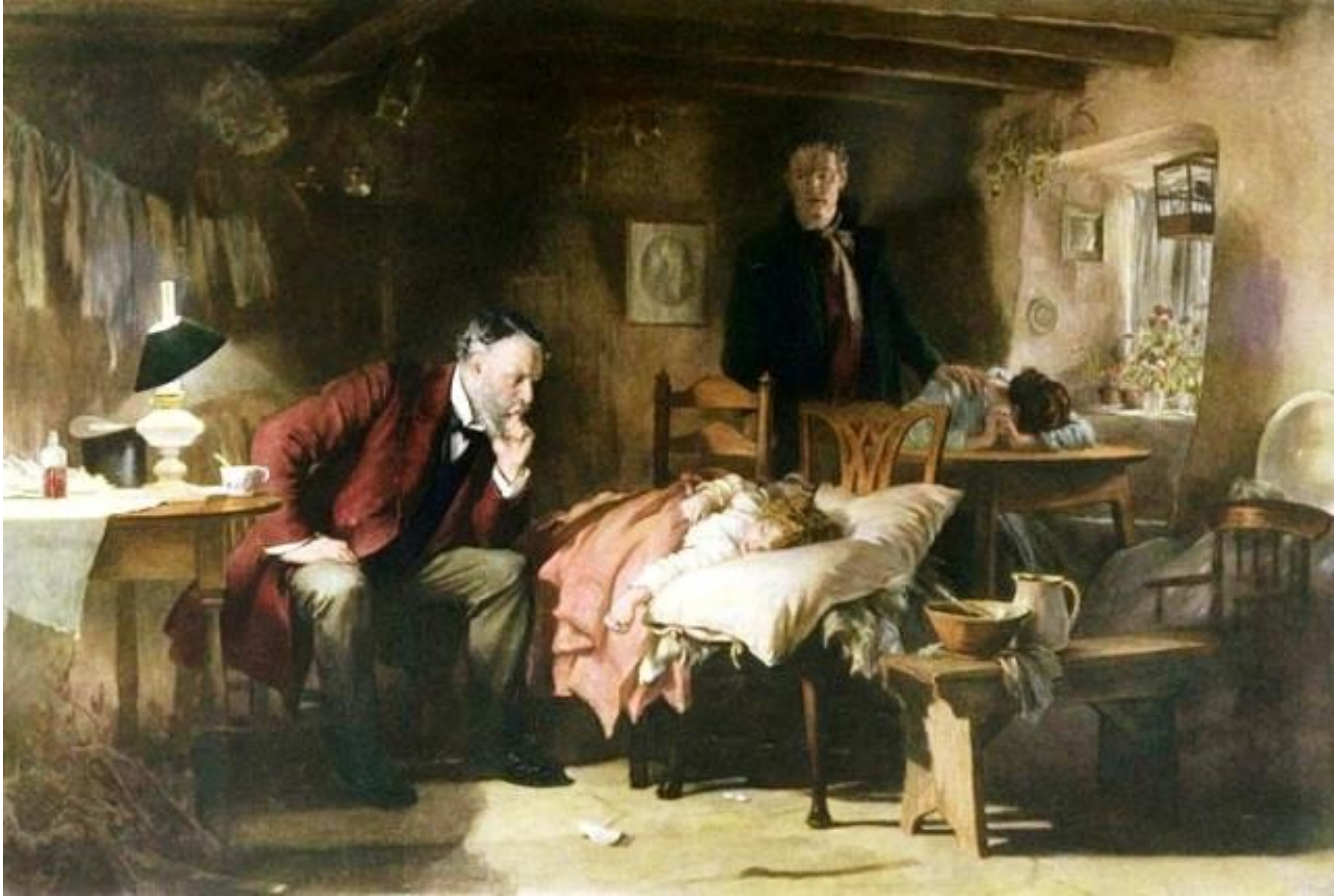

Self-understanding and interpretation of the painting

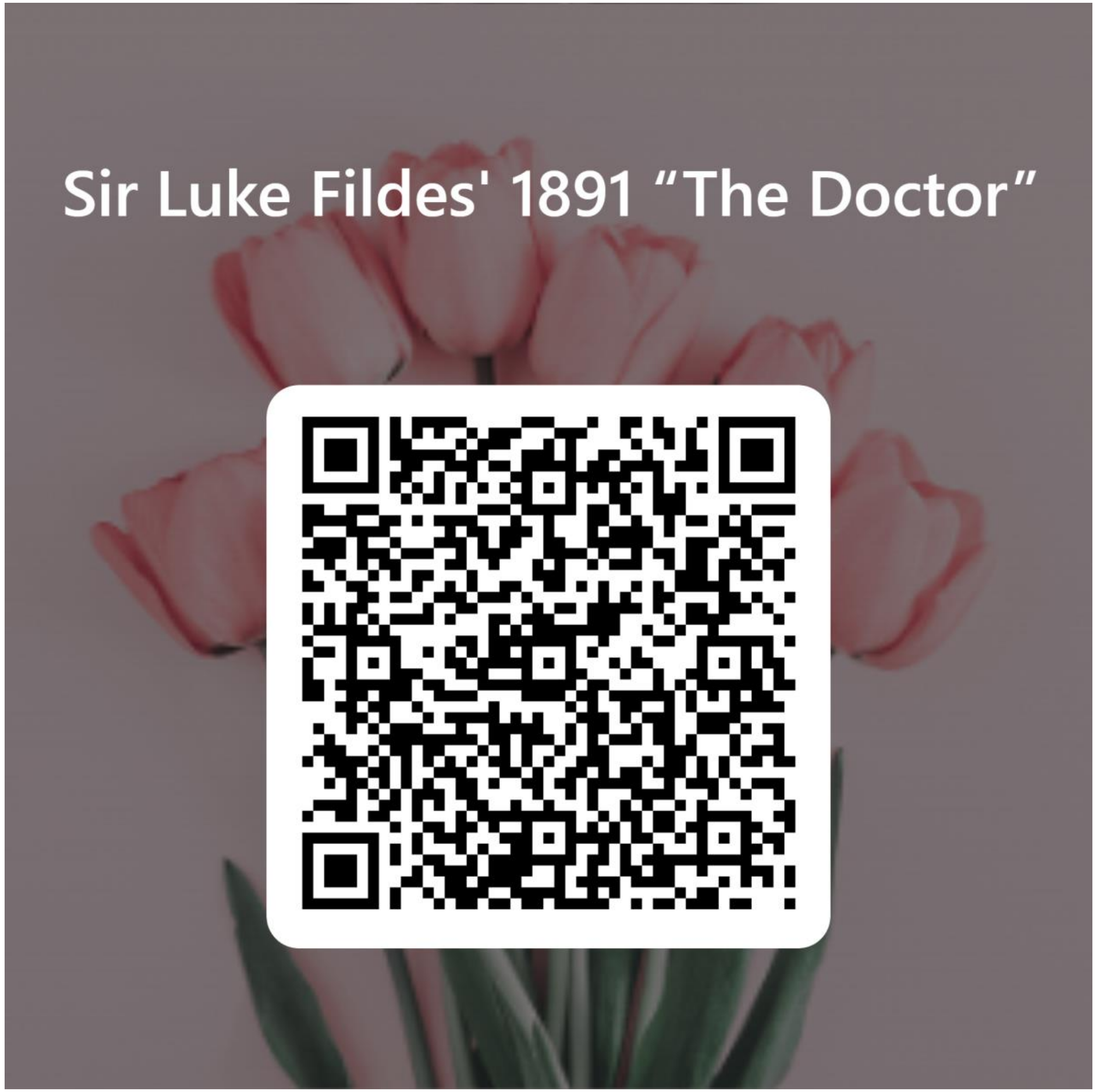

https://forms.office.com/r/gpbsN7dgLu

THINK PAIR SHARE

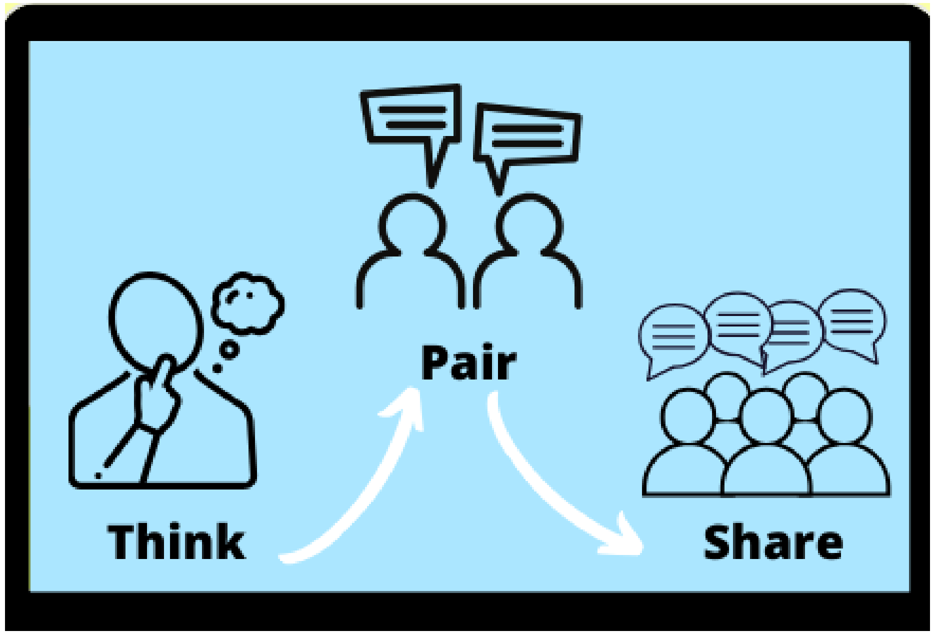

Introduction to the painting:
Sir Luke Fildes’ 1891 “The Doctor”

"One of the essential qualities of the Physician is an Interest in Humanity, for the secret of the care of the patient, is in caring for the patient".

Doctor’s Roles

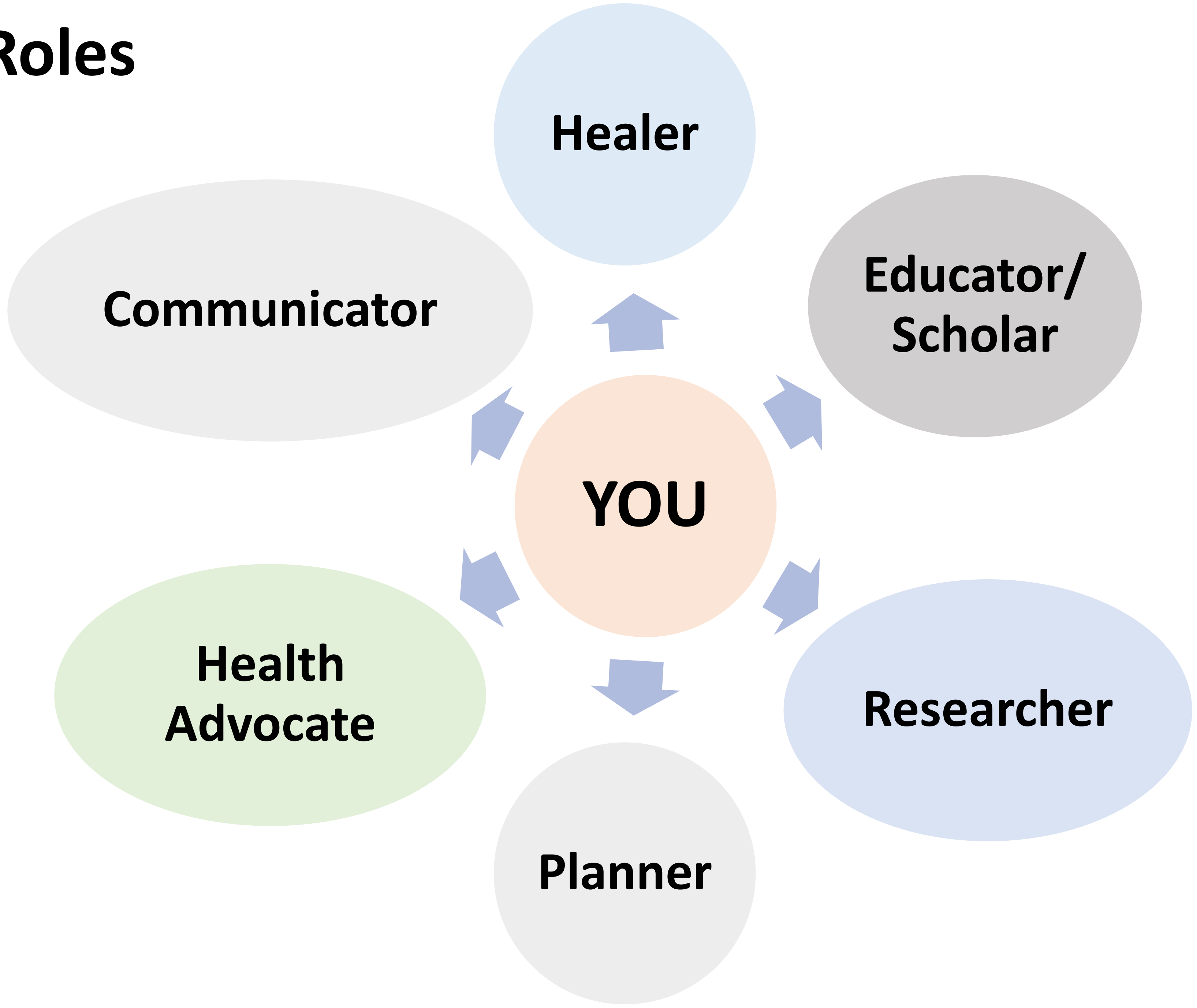

The Benefits of Empathy in Health Care

Better care

- ✓ Greater patient satisfaction
- ✓ Higher levels of hope
- ✓ Increased patient trust & ratings of clinical competence

Better workplace

- ✓ Improved provider well-being
- ✓ Greater job satisfaction
- ✓ Less burnout & absenteeism

Better health

- ✓ Improved medication adherence[1]
- ✓ Reduced pain[2]
- ✓ Reduced mortality[2]
- ✓ Fewer disease complications
- ✓ Improved immune function
- ✓ Decreased health care utilization & costs

[1]Meta-analysis
[2]Randomized controlled trials

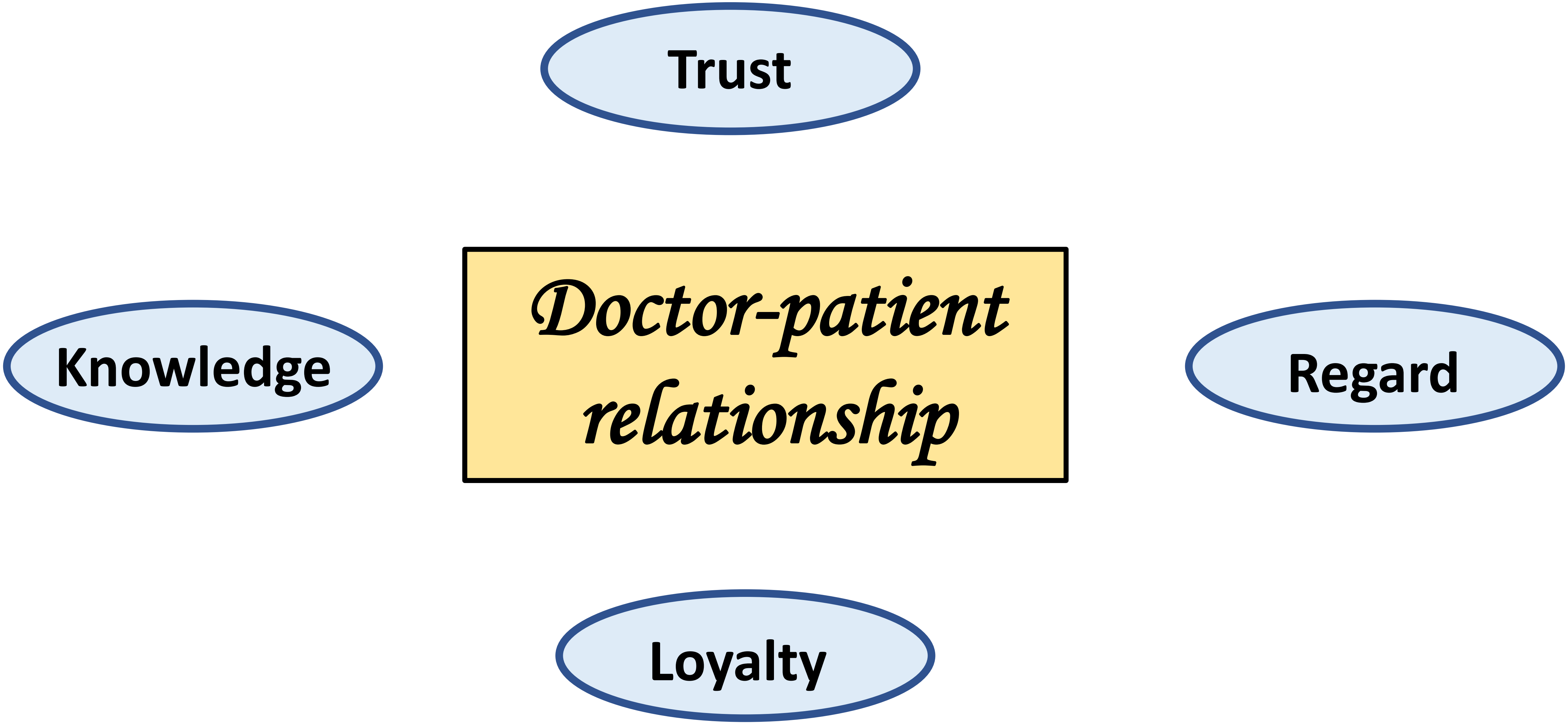

Prim Care Companion CNS Disord. 2015; 17(5): 10.4088/PCC.15f01840.
doi: 10.4088/PCC.15f01840

‘Trust’ in the doctor-patient relationship

“If a patient trusts you and feels like they can be honest with you without judgment, they'll be more likely to provide information that can help you provide better care”

- **Interpersonal trust & Social trust**

Patients reported trust as being substantially determined by their assessments of physician rapport, compassion, understanding, and honesty.
Trust in physician increases the likelihood of adhering to treatment recommendations.

When you put a patient's health and well-being above keeping down the health plan's costs **TRUST increases**…

Pearson SD, Raeke LH. Patients' trust in physicians: many theories, few measures, and little data. J Gen Intern Med. 2000 Jul;15(7):509-13. doi: 10.1046/j.1525-1497.2000.11002.

PATIENT'S RIGHTS

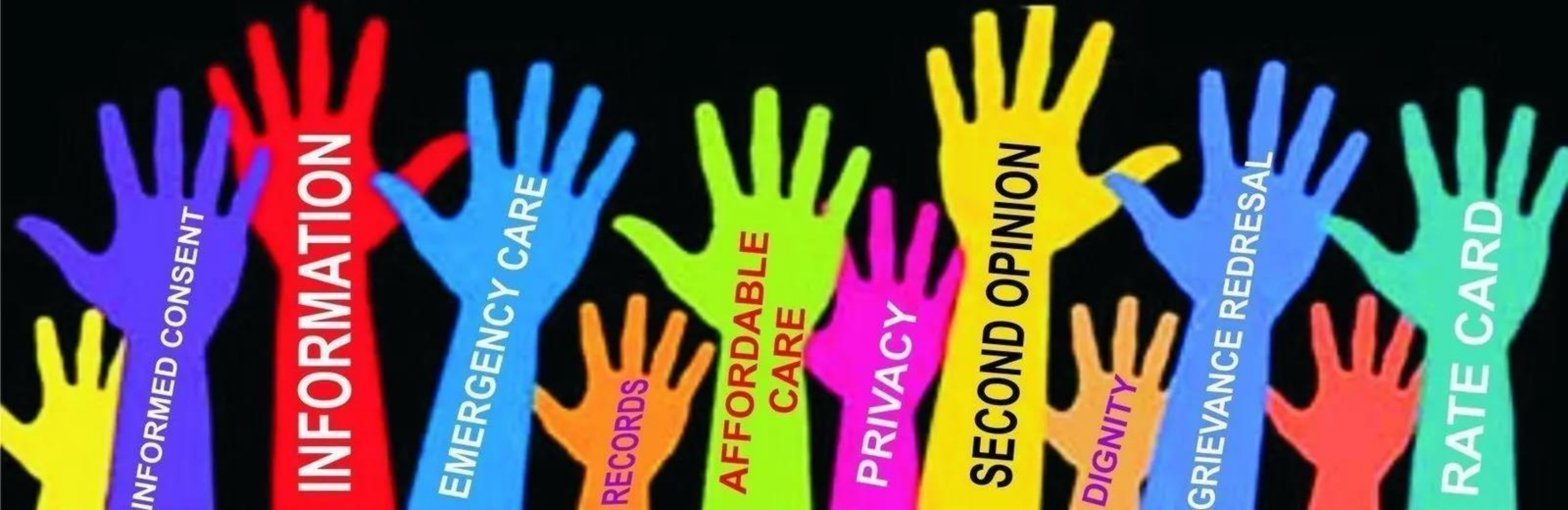

Rights of a Patient

Right to life and personal liberty:
Patients have the right to prompt emergency care by doctors without compromise on quality or safety and without having to pay full or an advanced fee to the hospital

- Right to Dignity and Privacy of Patient
- Right to ensure Safety
- Right to Confidentiality of Information
- Right to Refusal of treatment

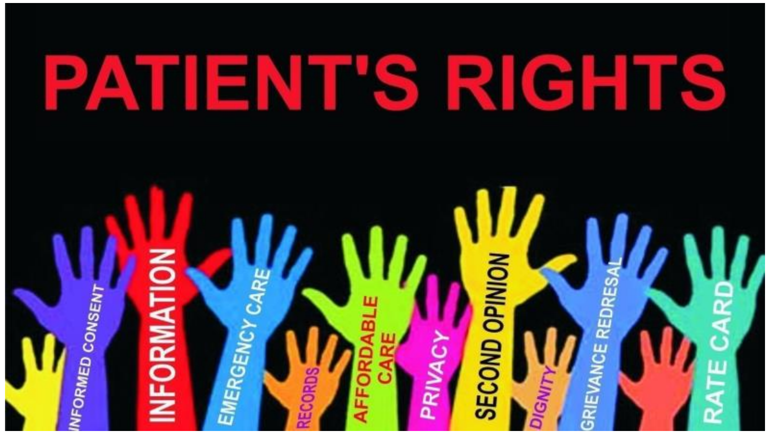

- To courtesy, respect, dignity, and timely responsive attention to his or her needs.
- To receive information from their physicians
- To have opportunity to discuss the benefits, risks, and costs of appropriate treatment alternatives, including the risks, benefits and costs of forgoing treatment.

Duties of a doctor

Knowledge, skills and performance

- Make the care of your patient your first concern
- Provide a good standard of practice and care
- Keep your professional knowledge and skills up to date
- Recognize and work within the limits of your competence

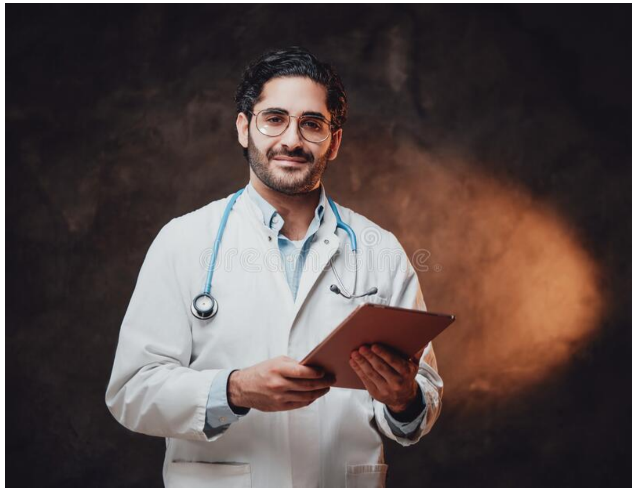

Safety and quality

- Take prompt action if you think that patient safety, dignity or comfort is being compromised
- Protect and promote the health of patients and the public

https://www.gmc-uk.org/ethical-guidance/ethical-guidance-for-doctors/good-medical-practice/duties-of-a-doctor

Duties of a doctor

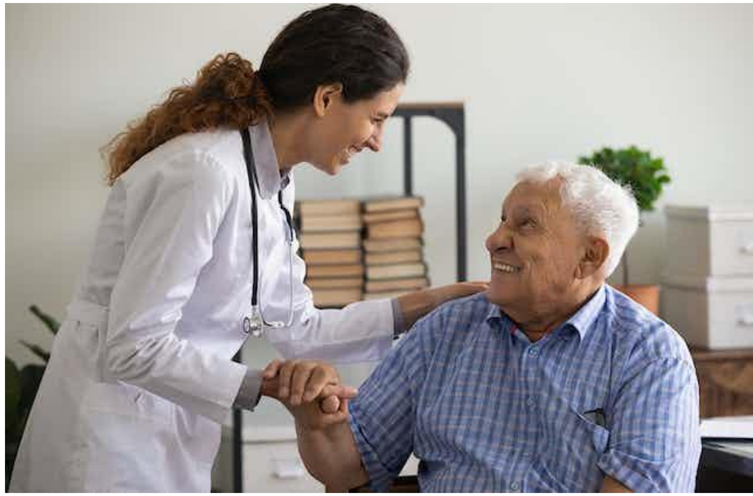

Communication, partnership and teamwork

- Treat patients as individuals and respect their dignity
- Treat patients politely and considerately
- Respect patients' right to confidentiality
- Work in partnership with patients
- Listen to, and respond to, their concerns and preferences
- Give patients the information they want or need in a way they can understand
- Respect patients' right to reach decisions about their treatment and care
- Support patients in caring for themselves to improve & maintain their health
- Work with colleagues in the ways that best serve patients' interests

https://www.gmc-uk.org/ethical-guidance/ethical-guidance-for-doctors/good-medical-practice/duties-of-a-doctor

Duties of a doctor

Maintaining trust

- Be honest and open and act with integrity
- Never discriminate unfairly against patients or colleagues
- Never abuse your patients' trust in you or the public's trust in the profession
- You are personally accountable for your professional practice and must always be prepared to justify your decisions and actions

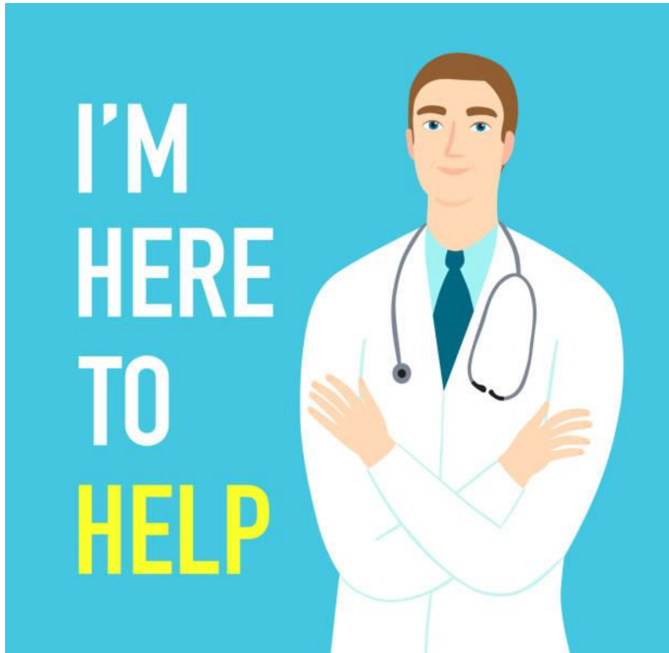

https://www.gmc-uk.org/ethical-guidance/ethical-guidance-for-doctors/good-medical-practice/duties-of-a-doctor

Boundaries in the doctor-patient relationship

- Business transactions
- Gifts and services
- Mishandling of fees
- Misuse of the physical examination

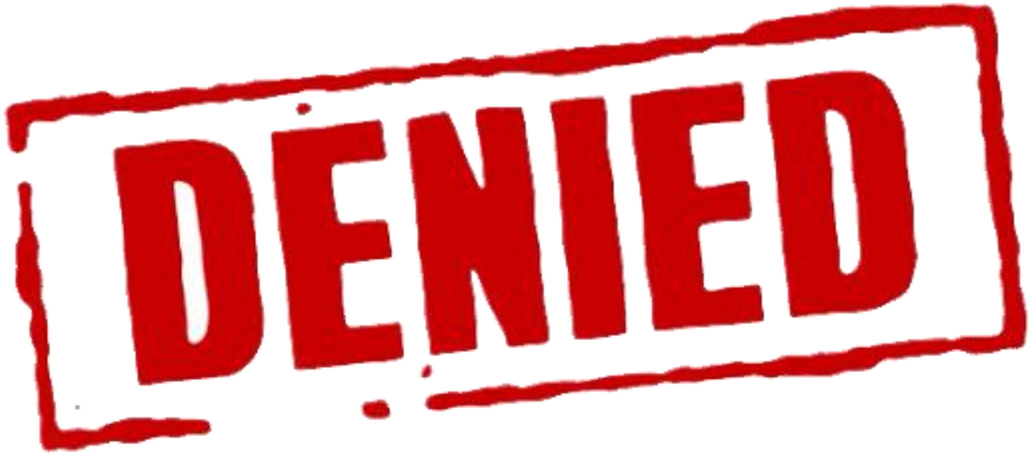

Boundary 'crossings' rather than violations and can sometimes be helpful.
Eg: For a physician to hold the hand of a patient who has lost a family member can be comforting, despite the fact that it counters usual recommendations about touching outside of the physical exam

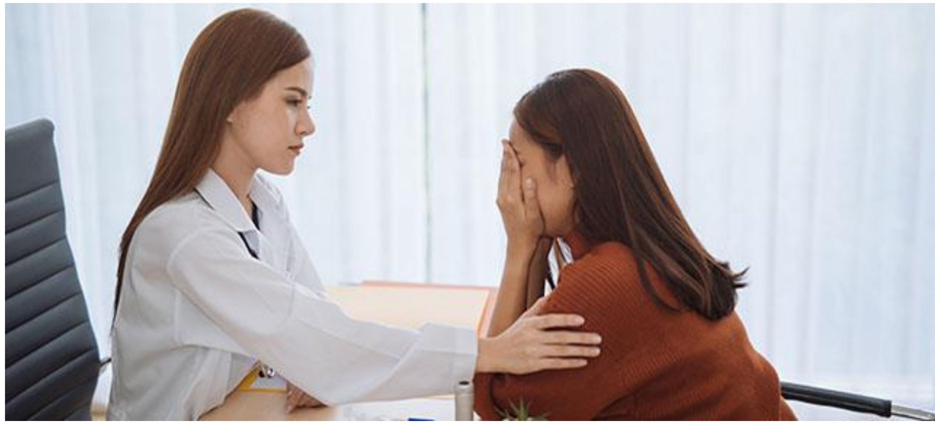

Nadelson C, Notman MT. Boundaries in the doctor-patient relationship. Theor Med Bioeth. 2002;23(3):191-201. doi: 10.1023/a:1020899425668.

Session evaluation 1

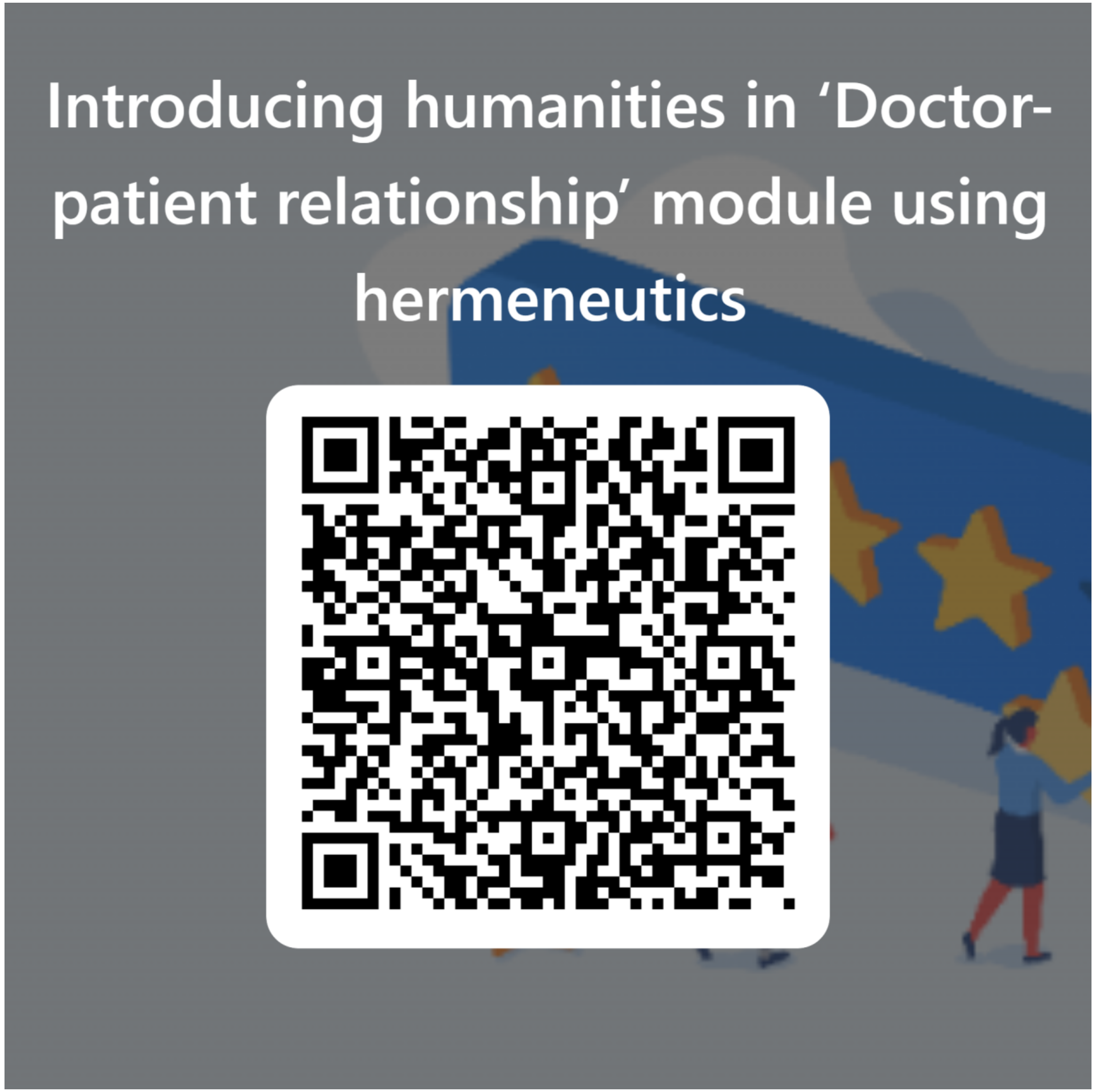

https://forms.office.com/r/2GqiEAz3qJ

Session evaluation 2

Reflective writing

1. Describe what happened?

2. What did I learn:
   - -Self-understanding & reflections about the painting
   - -‘Trust in the doctor-patient relationship’
   - -‘Rights of a patient and, Duties of a doctor
   - -‘Boundaries in the doctor-patient relationship’

3. The learnings from this session that I wish to apply in my future role as a healthcare provider or caregiver

PLEASE NOTE: UPLOAD THE REFLECTIONS IN Brightspace LMS
