# Supplementary material for: Visual hermeneutics as a tool to introduce empathy and core physician attributes in doctor-patient relationship for first-year medical undergraduate students
Source: BMC Med Educ. 2025 Jan 29;25:145. doi: 10.1186/s12909-025-06742-6 (PMC11780788; doi:10.1186/s12909-025-06742-6)
Supplement: Supplementary file 4 — Supplementary Material 4 [file 12909_2025_6742_MOESM4_ESM.pdf]

Kasturba Medical College and Kasturba Hospital
Institutional Ethics Committee

(Registration No. ECR/146/Inst/KA/2013/RR-19)
(DHR Registration No. EC/NEW/INST/2019/374)

Communication of the decision of the Institutional Ethics Committee

Wednesday 15th March 2023 **IEC1 : 80/2023**

Project title	:	"Being a Humanistic Doctor" Introducing Humanities in 'Doctor-Patient Relationship' Module for First-Year Medical Undergraduates using Hermeneutics.
Principal Investigator	:	Dr. Sushma Prabhath
Co Investigators	:	Dr. Uma Kulkarni, Dr. Kirtana R Nayak, Dr. Eshwari K, Dr. Divya Arvind Prabhu
Name & Address of Institution	:	Department of Anatomy, Kasturba Medical College, Manipal, Department of Ophthalmology, Yenepoya Medical College, Yenepoya University, Mangalore, Department of Physiology & Head of Department of Medical Education, Kasturba Medical College Manipal, Department of Community Medicine, Kasturba Medical College, MAHE, Manipal,
Status of review	:	New
Date of review	:	14.03.2023
Decision of the IEC	:	Approved with modifications till 31.07.2024.
IEC Approval Date	:	27 JUL 2023

Members present at the meeting

Dr. Ravindranath Rao (Chairperson & Social Scientist)
Mr. Rajaram Rao(Lay Person)
Dr. Mahadev Rao (Scientific Member)
Dr. Krishna Prasad P R (Clinician)
Dr. Sneha Deepak Mallya (Clinician)
Dr. Varalakshmi Chandra Sekaran (Clinician)
Dr. Vijetha Shenoy Belle (Clinician)
Dr. Sangita G Kamath (Basic Medical Scientist)
Dr. Vinu Thomas George (Scientific Member)
Dr. Saadi Abdul Vahab (Scientific Member)
Ms. Anupama (Legal Expert)
Dr. Binil (Scientific Member)
Dr. Arul Amuthan L(Basic Medical Scientist)
Dr. Sreenivasa Acharya (Social Scientist)
Dr. Rajeshkrishna Bhandary P (Member Secretary)

Standing Instructions

* The PI and all members of the project shall ensure compliance to current regulatory provisions (NDCT Rules 2019 and ICH-GCP), Ethical Guidelines for Biomedical Research on Human Participants by ICMR, and the SOP of IEC including timely submission of Interim Annual Report and Final Closure Report

* Participant Information Sheet and a copy of signed Informed Consent shall be given to every research participant (for all prospective studies)

* Inform IEC in case of any proposed amendments (change in protocol / procedure, site / Investigator etc.)

* Inform IEC immediately in case of any Adverse Events and Serious Adverse Events.

* Members of IEC have the right to monitor any project with prior intimation.

* **If CTRI/ HMSC/ CDSCO registration:** Ensure registration and clearance from the respective authorities before the enrollment of the first participant The IEC to be notified about the same within 7 days of successful registration/ clearance.

KMC & KHIEC APPROVED MANIPAL

Dr. Rajeshkrishna Bhandary P
MEMBER SECRETARY – KMC & KH IEC

(SOP: The chairperson endorses the comments before communicating to the investigators. The Member Secretary signs on behalf of the Chairperson on the IEC certificate)
