# Supplementary material for: Visual hermeneutics as a tool to introduce empathy and core physician attributes in doctor-patient relationship for first-year medical undergraduate students
Source: BMC Med Educ. 2025 Jan 29;25:145. doi: 10.1186/s12909-025-06742-6 (PMC11780788; doi:10.1186/s12909-025-06742-6)
Supplement: Supplementary file 5 — Supplementary Material 5 [file 12909_2025_6742_MOESM5_ESM.pdf]

Sir Luke Fildes' 1891 “The Doctor”

Sir Luke Fildes' 1887 “The Doctor”: Self-understanding and interpretation of the painting

Registration Number:

Gender: Male; Female; Others

Your thoughts and understanding about the painting:
