# Supplementary material for: Visual hermeneutics as a tool to introduce empathy and core physician attributes in doctor-patient relationship for first-year medical undergraduate students
Source: BMC Med Educ. 2025 Jan 29;25:145. doi: 10.1186/s12909-025-06742-6 (PMC11780788; doi:10.1186/s12909-025-06742-6)
Supplement: Supplementary file 6 — Supplementary Material 6 [file 12909_2025_6742_MOESM6_ESM.pdf]

To be included in Microsoft forms

Title of the study: Introducing humanities in 'Doctor-patient relationship' module for first year medical undergraduates using hermeneutics

Background: Hermeneutics can be employed in our efforts to introduce humanities in the "Doctor-patient relationship "module for first-year medical undergraduates. As a part of hermeneutics, Sir Luke Fildes' famous painting, "The Doctor" (1887, The Tate Britain, London), may be used to introduce humanities in the "Doctor-patient relationship" module for first-year medical undergraduates. It would be a first attempt wherein the interpretations made may create positive affirmations about medical humanities among the students and enhance their learning. It may further aid in imbibing the virtue of empathy and goodness in them toward patients right in their growing years as competent Indian Medical Graduates. The feedback obtained from the questionnaire will help us analyze the effectiveness of the session.

I understand that my participation in this questionnaire-based study is voluntary, and I can decline participation without giving any reason. I have been given sufficient time to review the information and sought the required clarification. I understand that I am allowed to take a screenshot of the form for my reference

Name of the research participant: ____

By clicking on the "I Agree" button, I consent to be a part of the study.

[I Agree] → Next to Questionnaire Page.

Demographic details:

Registration number:

Gender: Male, female, others (specify)

Age:

		Strongly agree	Agree	Neither agree nor disagree	Disagree	Strongly disagree
1	The session helped me understand the role of humanities in a doctor-patient relationship					
2	The image/painting chosen for the session evoked my interest					

3	The understanding of the image/painting provided by the facilitator was adequate					
4	The understanding of the image/painting provided by the facilitator was effective					
5	The presentation style of the facilitator was effective					
6	There was adequate time for discussions/interactions					
7	The venue chosen for the session was appropriate					
8	The logistics used during the session were adequate					
9	The duration of the entire session was adequate					
10	I enjoyed attending the session					
11	I actively participated in the interactive discussions					
12	I wish to apply this session's learnings in my future role as a healthcare provider.					
13	I liked the change of having this new teaching-learning method in the AETCOM session					
14	I wish to have such innovative teaching-learning methods in the future AETCOM sessions.					
15	I will recommend this learning experience to my peers					
16	How will you rate the overall learning experience?					

To be included in Microsoft forms

17	What did I like about the session?	
18	What could have been better OR can be further improved in the session?	
