# Supplementary material for: Visual hermeneutics as a tool to introduce empathy and core physician attributes in doctor-patient relationship for first-year medical undergraduate students
Source: BMC Med Educ. 2025 Jan 29;25:145. doi: 10.1186/s12909-025-06742-6 (PMC11780788; doi:10.1186/s12909-025-06742-6)
Supplement: Supplementary file 7 — Supplementary Material 7 [file 12909_2025_6742_MOESM7_ESM.pdf]

Critical appraisal of the Questionnaire for assessing the usefulness of 'Introducing Humanities in 'Doctor-Patient Relationship' Module for First-Year Medical Undergraduates using Hermeneutics'

BACKGROUND AND PURPOSE OF THIS QUESTIONNAIRE

Hermeneutics can be employed in our efforts to introduce humanities in the "Doctor-patient relationship "module for first-year medical undergraduates. As a part of hermeneutics, Sir Luke Fildes' famous painting, "The Doctor" (1887, The Tate Britain, London), may be used to introduce humanities in the "Doctor-patient relationship" module for first-year medical undergraduates. It would be a first attempt wherein the interpretations made may create positive affirmations about medical humanities among the students and enhance their learning. It may further aid in imbibing the virtue of empathy and goodness in them toward patients right in their growing years as competent Indian Medical Graduates. The feedback obtained from the questionnaire will help us analyze the effectiveness of the session.

INSTRUCTIONS FOR VALIDATING THE QUESTIONNAIRE:

This tool has 15 questions

Kindly review this tool and provide your feedback on the following:

The relevance of each question in the tool (how important is the question)
The clarity of each question (how clear is the wording)
The essentiality of each question (how necessary is the question)
Recommendations for improvement of each question

If you have any comments or correction feedback on the questionnaire **please make them on the copy provided.**

Please complete the following questions:

Name& Designation:
Department and Institute:
Academic experience in no of years:
Are you a member of MEU/Centre for Bioethics at your college? Do you have previous experience working with MEU/ Centre for Bioethics? If yes, how many years. (experience-5 years)
Any qualifications in medical education/ Bioethics?

1

Relevant Scale: 1= Not relevant; 2 = somewhat relevant; 3 = Quite relevant; 4 = Very relevant
Clarity Scale: 1= Not clear; 2 = Item needs some revision; 3 = Very clear
Essential Scale: 1 = Not essential; 2 = Useful but not essential; 3 = Essential

**Section One** (Fill in with color the relevant box)										
	**How relevant is this item?**				**Is this item clear?**			**How essential is this item?**		
Q.1	1	2	3	4	1	2	3	1	2	3
Q.2	1	2	3	4	1	2	3	1	2	3
Q.3	1	2	3	4	1	2	3	1	2	3
Q.4	1	2	3	4	1	2	3	1	2	3
Q.5	1	2	3	4	1	2	3	1	2	3
Q.6	1	2	3	4	1	2	3	1	2	3
Q.7	1	2	3	4	1	2	3	1	2	3
Q.8	1	2	3	4	1	2	3	1	2	3
Q.9	1	2	3	4	1	2	3	1	2	3
Q.10	1	2	3	4	1	2	3	1	2	3
Q.11	1	2	3	4	1	2	3	1	2	3
Q.12	1	2	3	4	1	2	3	1	2	3
Q.13	1	2	3	4	1	2	3	1	2	3
Q.14	1	2	3	4	1	2	3	1	2	3
Q.15	1	2	3	4	1	2	3	1	2	3

Questionnaire for assessing the usefulness of 'Introducing Humanities in 'Doctor-Patient Relationship' Module for First-Year Medical Undergraduates using Hermeneutics'

Sl. No.	Questions	Strongly agree	Agree	Neutral	Disagree	Strongly disagree
1	The session helped me understand humanities in a doctor-patient relationship					
2	The session accommodated my learning style					
3	The image "The doctor" chosen for the session was compelling					
4	The session was boring and monotonous					
5	The interpretation of the image was effective and adequate					
6	The presentation style of the facilitator was effective					
7	The interactions/ discussions					

	during the session were inadequate					
8	The venue chosen for the session was appropriate					
9	The logistics/resources used during the session was adequate					
10	The duration of the session was adequate					
11	Attending the session was worth my time					
12	I was unable to actively participate in the interactive discussions					
13	I wish to apply this session's learnings in my future role as a healthcare provider.					
14	What did I like about the session?					
15	What could have been better OR can be further improved in the session?					

RECOMMENDATIONS/COMMENTS/IMPROVEMENTS (Add your comments here)

1	The session helped me understand humanities in a doctor-patient relationship	
2	The session accommodated my learning style	
3	The image "The doctor" chosen for the session was compelling	
4	The session was boring and monotonous	
5	The interpretation of the image was effective and adequate	
6	The presentation style of the facilitator was effective	
7	The interactions/ discussions during the session were inadequate	
8	The venue chosen for the session was appropriate	
9	The logistics/resources used during the session was adequate	
10	The duration of the session was adequate	
11	Attending the session was worth my time	
12	I was unable to actively participate in the interactive discussions	
13	I wish to apply this session's learnings in my future role as a healthcare provider.	
14	What did I like about the session?	
15.	What could have been better OR can be further improved in the session?	

Feedback:
