# Supplementary material for: Visual hermeneutics as a tool to introduce empathy and core physician attributes in doctor-patient relationship for first-year medical undergraduate students
Source: BMC Med Educ. 2025 Jan 29;25:145. doi: 10.1186/s12909-025-06742-6 (PMC11780788; doi:10.1186/s12909-025-06742-6)
Supplement: Supplementary file 8 — Supplementary Material 8 [file 12909_2025_6742_MOESM8_ESM.pdf]

Email - Sushma Prabhath [MAHE | Edit Assignment - Anatomy | Preview Rubric: Rubric for asses
lighthouse.manipal.edu/d2l/lp/rubrics/preview.d2l?rubricId=941&originTool=rubrics&ou=17234
MeSH Browser | Yahoo India | Indexing and Archiv... | Irish Journal of Med... | Instructions for Aut... | Editorial Manager® | Journal of Histolog... | Authors Guidelines-... | All Bookmarks

Preview Rubric

Rubric for assessing Reflective Writing

Print

Criteria	Level 5<br>5 points	Level 4<br>4 points	Level 3<br>3 points	Level 2<br>2 points	Level 1<br>1 point	Criterion Score
Reflective Thinking	Reflection clearly explains the student's own thinking about his/her own learning processes, as well as implications for future learning	Reflection clearly explains the student's own thinking about his/her own learning processes.	Reflection attempts to demonstrate thinking about learning and has some clarity about the personal learning process	Reflection attempts to demonstrate thinking about learning but is vague and/or unclear about the personal learning process.	Reflection does not address the student's thinking and/or learning.	/ 5

Type here to search | 30°C Mostly sunny | ENG | 01:35 PM 14/10/2023

Email - Sushma Prabhath [MAHE | Edit Assignment - Anatomy | Preview Rubric: Rubric for asses
lighthouse.manipal.edu/d2l/lp/rubrics/preview.d2l?rubricId=941&originTool=rubrics&ou=17234
MeSH Browser | Yahoo India | Indexing and Archiv... | Irish Journal of Med... | Instructions for Aut... | Editorial Manager® | Journal of Histolog... | Authors Guidelines-... | All Bookmarks

Analysis	Reflection is an in-depth analysis of the learning experience, the value of the derived learning to self or others, and the enhancement of the student's appreciation for the discipline. Includes supporting details and examples.	Reflection is an analysis of the learning experience and the value of the derived learning to self or others. Includes some supporting details and examples.	Reflection attempts to analyze the learning experience with some value of the learning to the student or others Includes a few supporting details and examples.	Reflection attempts to analyze the learning experience but the value of the learning to the student or others is vague and/or unclear. Includes a few supporting details and examples	Reflection does not move beyond a description of the learning experience. There are no details.	/ 5
Quality of Information	Information clearly relates to the main topic. It includes several supporting details and/or examples.	Information clearly relates to the main topic. It provides 2-3 supporting details and/or examples.	Information clearly relates to the main topic. It provides only one supporting detail and/or example.	Information clearly relates to the main topic. However, no details and/or examples are given.	Information has little to do with the main topic.	/ 5
Structure & Organization	Writing is clear, concise, and well organized with the use of excellent	Writing is clear, concise, and organized with the use of excellent	Writing is mostly clear, concise, organized with the use of good	Writing is unclear, and thoughts are vaguely organized. Thoughts are not	Writing unclear, disorganized. Thoughts make little to no sense making it	/ 5

Type here to search
30°C Mostly sunny ENG 01:35 PM 14/10/2023

Email - Sushma Prabhath [MAHE | Edit Assignment - Anatomy | Preview Rubric: Rubric for asses

lighthouse.manipal.edu/d2l/lp/rubrics/preview.d2l?rubricId=941&originTool=rubrics&ou=17234

MeSH Browser | Yahoo India | Indexing and Archiv... | Irish Journal of Med... | Instructions for Aut... | Editorial Manager® | Journal of Histolog... | Authors Guidelines-... | All Bookmarks

	examples.		example.			
Structure & Organization	Writing is clear, concise, and well organized with the use of excellent sentence/paragraph structure. Thoughts are expressed in a logical manner and makes understanding easy.	Writing is clear, concise, and organized with the use of excellent sentence/paragraph structure. Thoughts are mostly expressed in a logical manner and contributes to easy understanding.	Writing is mostly clear, concise, organized with the use of good sentence/paragraph structure. Thoughts are mostly expressed in a logical manner and contributes to easy understanding	Writing is unclear, and thoughts are vaguely organized. Thoughts are not expressed in a logical manner and this impedes the understanding.	Writing unclear, disorganized. Thoughts make little to no sense making it extremely difficult to understand	/ 5

Total	/ 20

Overall Score

Overall Feedback
0 points minimum

Type here to search | 30°C Mostly sunny | ENG | 01:36 PM 14/10/2023
