# Supplementary material for: Visual hermeneutics as a tool to introduce empathy and core physician attributes in doctor-patient relationship for first-year medical undergraduate students
Source: BMC Med Educ. 2025 Jan 29;25:145. doi: 10.1186/s12909-025-06742-6 (PMC11780788; doi:10.1186/s12909-025-06742-6)
Supplement: Supplementary file 9 — Supplementary Material 9 [file 12909_2025_6742_MOESM9_ESM.pdf]

[redacted]

Date: ....../....../......

AETCOM Session
Reflective Writing

1. Describe what happened?

Ans - A discussion was done on a very elaborate & informative painting.
- Many ~~informative~~ important topics were conveyed this way.
- Then, there was a discussion on the duties of a doctor & what exactly they mean.
- Further we learnt the meaning & importance of empathy. Finally, we learnt about the rights of a patient & duties of a doctor.

2. What did I learn?

Ans - The painting conveyed the compassion & empathy of the doctor. He provided the best care possible to the patient.
- Trust is important in the doctor-patient relationship b/c if a patient trusts the doctor, he is more likely to follow the medication & his stress is also lowered.
- Patients have the right to emergency treatment, without paying beforehand.
- They have the right to refuse treatment
- Doctors must be empathic & provide the best care possible. They must respect the boundaries of the patient.
- Doctors must take consent before doing anything to the patient.

[illegible]

Date: ....../....../..........

Duties of a doctor :- Healer
- Communicator
- Planner
- Researchers
- Educator / Scholar
- Health Advocate

3. The learnings from this session that I wish to apply in my future role as a healthcare provider or care giver.

Ans - I will try to have empathy for all patients.
- I will respect personal boundaries.
- I will ensure that my communication is clear & easy to grasp.
- I will consider it my responsibility to educate the public about health & medicine.

Session evaluation – 2

Reflective writing

Q1. Describe what happened?

The session was on doctor patient relationship. We were shown a painting on the above topic. We discussed about the painting. Then there was presentation on the same topic.

Q2. What did I learn

- Painting depicted a doctor who is very patient and dedicated to his job. He with the minimum supplies he had, ~~[illegible]~~ did his maximum to help the child.
- Trust in doctor-patient relationship is very important. Only then the patient will adhere to the medications prescribed and come for follow up check ups. It help in faster, better recovery of patient.

- Rights of patients, duties of doctor

The patients have right to information, autonomy over medical procedures, treatment without discrimination, privacy, safe and quality treatment.

As doctors we have to treat everyone fairly, with respect respect their privacy, provide the best of your knowledge and skills needed for condition and not cause any harm to patient.

Boundaries in doctor patient relationship.
We have to treat patients as much as we can and then refer them to specialists for more treatment. There should be mutual respect and honesty in the relationship.

3. The learnings from session that I wish to apply in my future role as a healthcare provider or caregiver

Good communication and establish trust with my patients.
To avoid medical jargons.
To take care of them as much as my best ability and knowledge.

Page

Q1, Describe what happened ?
We had an AETCOM session over the topic 'Doctor - Patient relationship' in which we are shown a painting by sir Luke Fildes named 'The Doctor' which came out in 1891.
We were asked to provide a self interpretation of the painting, after which we had a small discussion about our personal thoughts about the painting & then teacher discussed about painter's view over his painting.
We were informed how painter depicted various roles & qualities of good doctor & rights of patients.

Q2, What did I learn ?
• Self understanding & reflections about the painting
My overall self understanding of painting was that it revolved around a sick girl & the doctor. The doctor seemed to be there since quit some time & girl's parents were present at back waiting for doctor to update them about the situation while doctor sat there trying to analyze the situation.

• Trust in doctor patient relationship
I learned that in a doctor - patient relationship, having trust on each other & communicating the problems properly plays a great role. Not holding back even the slightest detail is extremely important. It is also to be kept in mind

that it becomes doctor's responsiblity when their patient shows trust in them to not only treat the patient but also understand their conditions & circumstances (financially, emotionally, physically & mentally.)

Right of a patient & duties of a doctor

I learned about various rights of a patient such as their right to access affordable health care, right to information etc as well as duties of doctor towards their patient & how an action or jesture can hold diff meaning in diff situations.

Boundaries in doctor patient relationship

I learned that even though boundaries are imp, consoling patient & their family might be appropriate in some situation, though their comfort must be considered first.

Q3. The learning from this session that I wish to apply in future
⇒ giving my undivided attention to the patient I am attending at that moment; non considering financial benefits & valuing patient's interest; being professional while not losing empathy.

Session evaluation 2

① Describe what happened?

The Doctor is an 1891 painting by Luke Flides that depicts a Victorian doctor observing the critical stage in a child's illness while the parents gaze on helplessly from the periphery. It has been used to portray the values of the ideal physician and the inadequacies of the medical profession. Different theories exist as to the painting's ~~design~~ origin but it is most likely based upon Flides' own experience of the death of his son. Critics have noted that Flides omitted common medical equipment of his era in order to focus on the relationship between physician and patient.

② What did I learn?

- To have faith in the doctor
- To respect the patient and vice versa.
- Patient's right to know his condition
- Patient's right to know his treatment options.
- Doctor's duty to do right by ~~f~~ his patient
- To not cross lines beyond the doctor-patient relationship
- To have trust in the doctor-patient relationship

DOMS

___/___/___

(3) Learnings that I wish to apply in my future role as a healthcare provider.

- → Great communication skills.
- → Keen listening quality
- → Decisivenes and Dedication
- → Trust and Respect
- → Honesty and appropriate treatment
- → No cheating / bribing / hiding from the patient
- → Empathy and Friendliness.
- → Passion.
- → Observant, caring and Patient

—x—x—

EXPT. NO. | NAME | M T W T F S S | Page No.: | Date: | YOUVA

AETCOM

Reflective writing

Q1 Describe what happened?

Ans We were shown a painting and we discussed each other's interpretation of the painting. This was a new way of learning which helped us in learning many qualities of a doctor.

Q2 What did I learn?

Ans) Self understanding and reflections about painting

i) Doctor exhibits patience
ii) Doctor was focussed on his duties

Trust in the doctor patient relationship

i) prevents 2° manifestation
ii) Ensure better medicine

Rights of pat[illegible] [illegible]r

i) RTI
ii) Respect the [illegible]

Q3) Learning fro[illegible] I wish to apply in my future [illegible] provider.

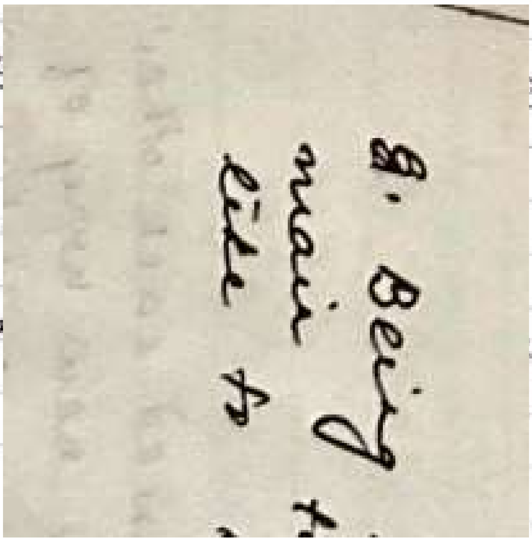

empathetic and high level of patience are
takeaways from the session that I would
implement.

SESSION EVALUATION-2.

Reflective writing.

1. Describe what happened?

2. What did I learn
   - Trust in doctor patient relationship
   - Rights of a patient & duties of a doctor
   - Boundaries in a doctor patient relationship.

3. The learning from this session that I wish to apply in my future role as a health care provider or caregiver.

1. The lecturers of our college were kind enough to schedule an AETCOM lecture for us where we learned something much different & much bigger than the usual textbook learning. The morals I gained today are always going to stay with me.

2. Today's session made me introspect myself and understand that being a doctor does not merely means curing a sick patient, rather healing an ailing individual. I understood the importance of building trust in a doctor patient relationship and how will I have to proceed with compassion and empathy.
As a doctor it is our duty to ensure the best treatment to the patient and respect the rights the patient has. These rights and duties are often neglected but their importance is what we learned and acknowledged today. Also while treating the patient we need to respect

Scanned with AnyScanner

the boundaries we set in the professional relationship. but sometimes we might feel the need to stape a step forward and beyond the boundary in order to provide comfort to the patient without crossing the boundary.
It obviously is a very sensitive topic but the perfect balance between it is what differentiates a doctor from a good doctor.

3. One thing I definately want to understand and implement in my life is to not just provide the treatment to the patient rather heal him with the empathy and compassion. As a doctor should see in a patient is a fellow creature in suffering and provide the appropriate treatment.

Scanned with AnyScanner

[illegible]

[illegible]

(1) Lecture began with the interpretation of the famous painting 'the doctor' by Filde. After that we learnt the duties of doctor and about doctor-patient relationship. The teacher calmly explained each point in detail & accurately.

(2) (a) I learnt how a doctor should behave and understand the patient, tending to his/her needs.

(b) very imp. as it helps the doctor to get to know more about the patient which the patient might not have revealed earlier.

(c) Patient has the right to information and to participate in decision-making.

(d) Doctor must take consent of the patient before examination. Not to touch the patient inappropriately. Not to take gifts.

(3) should be a good healer, compassionate, good planner, health advocate.
Must understand the intrinsic values of the patient.

_/_/_

AETCOM - DOCTOR - PATIENT RELATIONSHIPS

REFLECTIVE WRITING

Q1. Describe what happened:

Ans. We were shown a painting, 'The Doctor' by Luke Filde. Following which we were given time to interpret the painting. Then we discussed it all and correlated it to the painter's point of ~~vo~~ view. After which we learnt about Doctor - Patient Relationships.

Q2. What did I learn:

- Self Understanding & Reflections about the painting
  → The painting showed a doctor who was deep in thought about a patient. He was so lost in thought that he spent hours in the patient's house, despite the socio-economic difference. Meanwhile, the mother was very worried and the father was unable to console her as they did not know what would happen to their child.

- Trust in the doctor - patient relationship
  Patient trusts ~~the~~ doctor when the doctor uses right treatment regime, listens to the patients concerns,

sympathises, empathises, is compassionate, understanding and honest.

- Rights of a patient & Duties of a Doctor
  → Patient Rights are information, second opinion, affordable care, alternate treatment plan, emergency care, confidentiality.

  → Duties of doctor are healer, communication, planner, understanding compassion etc.

- Boundaries in doctor patient relationship
  → Necessary
  Unethical to accept gifts
  → Do not cross the line between private & personal life of the patient & doctor.
  → Sometimes necessary to break to help & console the patient.

Q3. The learnings from this session that I wish to apply in my future role as a healthcare provider or caregiver.

Ans. To be an empathetic doctor whose patients trust & regard him and who stays loyal to his patients. Also, to use my knowledge & skill set & to keep expanding my knowledge to provide patients with the best possible healthcare.

Date / /

AETCOM - DOCTOR- PATIENT RELATIONSHIP

* What did I learn?
  - → Trust in doctor-patient relationship
  - → Rights of patient & duties of a doctor
  - → Unwavering commitment of a doctor to fulfil his call of Duty

* The learning from this session that I wish to apply in my future role as a health care provider/caregiver

→ The lecturers of our college were kind enough to schedule an AETCOM lecture for us where we learnt something much different & much bigger than the routine learning

→ The values imparted by these sessions have to be inculcated by all of us in order to be successful physicians.

→ It has made me realise, what it actually means to 'heal' a patient

→ As a doctor, it is our duty to ensure the best possible treatment to the patient and respect the rights the patient has.

→ These rights and duties are often neglected but their importance is what we acknowledged today

→ Today's session has made me realise the importance of having an empathetic and compassionate attitude while treating a patient

→ We can provide the best possible service only when we stand in the patient's shoes

/ /

AETCOM - DOCTOR - PATIENT RELATIONSHIPS

REFLECTIVE WRITING

Q1. Describe what happened:

Ans. We were shown a painting, 'The Doctor' by Luke Filde. Following which we were given time to interpret the painting. Then we discussed it all and correlated it to the painter's point of ~~ve~~ view. After which we learnt about Doctor - Patient Relationships

Q2. What did I learn:

- Self Understanding & Reflections about the painting
  → The painting showed a doctor who was deep in thought about a patient. He was so lost in thought that he spent hours in the patient's house, despite the socio-economic difference. Meanwhile, the mother was very worried and the father was unable to console her as they did not know what would happen to their child.

- Trust in the doctor - patient relationship
  Patient trusts ~~the~~ doctor when the doctor uses right treatment regime, listens to the patients concerns,

_/_/_

sympathises, empathises, is compassionate, understanding and honest.

- Rights of a patient & Duties of a Doctor
  → Patient Rights are information, second opinion, affordable care, alternate treatment plan, emergency care, confidentiality.

  → Duties of doctor are healer, communication, planner, understanding compassion etc.

- Boundaries in doctor patient relationship
  → Necessary
  Unethical to accept gifts
  → Do not cross the line between private & personal life of the patient & doctor.
  → Sometimes necessary to break to help & console the patient.

Q3. The learnings from this session that I wish to apply in my future role as a healthcare provider & caregiver.

Ans. To be an empathetic doctor whose patients trust & regard him and who stays loyal to his patients. Also, to use my knowledge & skill set & to keep expanding my knowledge to provide patients with the best possible healthcare.

Date ________ Page ________

Reflective Writing:

1.- Describe what happened?.
Ans.- We learn about what is doctor role's. we also learn about duties of doctor and also learn about empathy to patients help in better medication adherence.

2.- What did I learn:
→ Self-understanding & Reflections about the paintaing. The paintaing showed doctor has tried all his medication trials and waiting for the out come on patient very patiently. So, this paintaing made me learn.
- Have patient.
- Show patient interest.
- Built trust.

→ 'Trust in the doctor-patient relationship'. Trust is the key factor in doctor-patient relationship because when we built trust in patient. Patient share his sufferings nicely. And also share his history properly.

→ Rights of a patient and, Duties of Doctor.
· Right of patient are-
Right to deny for treatment.
Right to take decision for their treatment.
Duties of Doctor are:-
- Proper provider for health medication
- Researcher.

'Boundaries in the doctor-patient relationship'

⇒ Do as much as knowledge you have and then refer the patient further to the respective specialist.

3.- The learnings from this session that I wish to apply in my future role as a healthcare provider or caregiver.

The learnings from this session which I will apply are -

- Always maintain a good relationship with patient.
- Communicate properly.
- Do not use medical terminology in front of patient and scare him out.
- Developing trust in patient so that he will follow up on your medication and come again to you.
- The duties of doctor which we have to do when we serve as healthcare provider.
- Respect the patient deny for treatment.

29 July 2023

[illegible]

S. No.: 247

Roll No [illegible]

Reflective Writing

1. Describe what happened?

A- We learnt about doctor-patient relationship by the means of understanding a patient whilst co-relating it with our lives.

2. What did I learn:

✓ Self understanding & reflections about the painting.

✓ Trust in doctor-patient relationship.

✓ Rights of a patient, and duties of a doctor.

✓ Boundaries in the doctor-patient relationship.

3. The learning's from this session that I wish to apply in my future role as a healthcare provider or caregiver.

A- 'Listen, build trust and heal.' I would ~~som~~ sum up today's session with those words. I learned about how I can touch lives and contribute my share in the challenges I'll be faced with one day.
Further, the element of being empathetic with the patient and its family is a pre-requisite of a healthcare provider/caregiver

[ACTCOM] 01|08|23

[Ques-1] Describe what happened?

[Ans.] Getting the basics of what is required in a doctor as a service provider in a form of interactive painting discussion surely proved to be an amazing experience. It was interesting & fruitful.

[Ques-2] What did I learn?

[Ans-] 1) self reflection & understanding about painting.

⇒ A doctor serves the needy irrespective of socio-economic barriers, if any.

While examining treating & post recuperation period, every time is crucial for the patient & his/her family. The doctor needs to be alert & attentive to the patient & provide an empathetic & caring environment.

2) Trust in doctor-patient relationship

Trust should be the brick to any doctor patient relationship.

This ensures a positive environment for the patient to be treated as well as a method of relief that the patient would religiously follow the prescribed treatment for their betterment.

3) Rights of a patient & duty of a doctor

⇒ The patient has the right to know the ifs & do's & don'ts related to their treatment.

The patient must be told about the risks & expenses involved in the procedures.

⇒ Its the duty of a doctor to explain everything clearly to the patient & their family beforehand

4) Boundaries in a doctor-patient relationship

⇒ To ensure an equal & just treatment for all the patients, there shouldn't be involvement of any kind of favours, be it in face of gifts or help of any kind.

⇒ The doctor must always treat the patients without any business & greedy interest involved.

Ques ③ Learnings from the session that I wish to apply in my future role as healthcare provider.

Ans. Empathy & care are truly the best form of support one can offer to the patient/family.
A positive outlook towards the procedure & treatment builds up trustworthy doctor-patient relationship.
There shouldn't be any involvement of any shady or greedy business involvements in treatment of the patient & all risk & expenses must be mentioned to the patient & family beforehand.

Session Evaluation 2

Reflective Writing

1. Describe what happened?
2. What did I learn
   - Self understanding and reflections about the painting
   - Trust in the doctor-patient relationship
   - Rights of a patient, Duties of a doctor
   - Boundaries in the doctor-patient relationship

3. The learnings from this session that I wish to apply in my future role as a healthcare provider or caregiver

Answers

1. Discussion of a painting 'The Doctor', and then the introduction of the concept of humanity in doctor patient relationship was done

2. a) The humane nature of the doctor, who was not hesistant to treat someone poor.
   b) Trust is v imp for a doctor
   c) The patient has certain rights to information, affordable care which the doctor cannot deny. Duties of [illegible] empathy are cruc[illegible]
   d) Bound[illegible] nd no unu[illegible] also essential [illegible]

Date ______
Page ______

[illegible] from this session I would want to apply

[illegible]ing respect to and from the patient
[illegible]hy
the patient his/her rights
[illegible]ng that the boundaries of the doctor-
relationship are maintained.
